# Agro-Industrial Biowaste Valorisation by Engineering Controlled-Release Polyphenol Products for Applications in Sustainable Agriculture

**DOI:** 10.3390/polym18060715

**Published:** 2026-03-16

**Authors:** Fabrizio De Cesare, Simone Serrecchia, Gabriella Di Carlo, Cristina Riccucci, Gianmarco Alfieri, Andrea Bellincontro, Sarai Agustin-Salazar, Gabriella Santagata, Paolo Papa, Antonella Macagnano

**Affiliations:** 1 Department for Innovation in Biological, AgriFood and Forest Systems (DIBAF), University of Tuscia, 01100 Viterbo, Italy; gian.alfieri@unitus.it (G.A.); bellin@unitus.it (A.B.); 2 Institute of Atmospheric Pollution Research (IIA), National Research Council (CNR), 00010 Montelibretti, Italy; simoneserrecchia@cnr.it (S.S.); paolo.papa@cnr.it (P.P.); 3 Department of Chemical, Materials and Industrial Production Engineering (DICMAPI), University of Naples Federico II, 80125 Naples, Italy; 4 Institute for the Study of Nanostructured Materials (ISMN), National Research Council (CNR), 00010 Montelibretti, Italy; gabriella.dicarlo@cnr.it (G.D.C.); cristina.riccucci@cnr.it (C.R.); 5 Institute of Polymers, Composites and Biomaterials (IPCB), National Research Council (CNR), 80078 Pozzuoli, Italy; sarai.agustinsalazar@cnr.it (S.A.-S.); gabriella.santagata@ipcb.cnr.it (G.S.)

**Keywords:** polyphenols, lignin, drug delivery, electrospinning, electrospraying, agro-industrial waste, polyhydroxybutyrate (PHB), polycaprolactone (PCL)

## Abstract

Electrospinning and electrospraying nanotechnologies were used to valorise agro-industrial residues into biohybrid controlled-release polyphenol (CRP) scaffolds. Four polyhydroxybutyrate ± polycaprolactone (PHB±PCL) architectures were fabricated that differed in polymer phase, Klason lignin from hazelnut shell (HS-KL) presence vs. absence, and co-location with grape-pomace polyphenols (GP-PPs), as well as in distribution between fibres and bead-like depots. Scaffolds were characterised using optical microscopy/stereomicroscopy/SEM, FTIR, UV–Vis spectroscopy, and dynamic water contact angle (absorption). GP-PP release was monitored for 14 days at ~25 °C and 37 °C, the latter representing shallow-soil hot-spell conditions in Mediterranean zones. All matrices exhibited multimodal release, with modest initial bursts and three phases (*burst*, *mid*, and *late tail*), analogous to controlled-release fertiliser profiles. At ~25 °C, the PHB/PCL matrix with HS-KL confined to PHB fibres and GP-PP in large PCL beads showed the highest *total* GP-PP release, whereas the architecture with HS-KL and GP-PP co-located in both PHB and PCL fibres and in PCL depots combined high *total* release with a smoother, well-metered late phase. At 37 °C, this HS-KL-GP-PP co-located scaffold was the most robust, retaining the highest *total* and *late tail* release. These results identify HS-KL-GP-PP co-located PHB/PCL architectures as promising carriers for temperature-resilient delivery of bioactive polyphenols in Mediterranean agrosystems.

## 1. Introduction

Increasing agricultural productivity is essential to sustain the growing global food demand [1]. This requires both improving crop yields and reducing losses along the production chain [2]. Unfortunately, many current strategies for boosting yields (from frequent fertiliser and pesticide applications to coated slow- and controlled-release products) still suffer from timing mismatches between nutrient or active-ingredient supply and plant demand, high off-target losses (leaching, volatilisation, and runoff) [3,4,5] and, in the case of polymer-coated granules, persistent plastic residues accumulating in soils [6]. There is, therefore, a need for bio-based delivery systems that can better synchronise release with crop requirements while avoiding long-lived synthetic coatings and minimising environmental burdens [7]. Materials science—and in particular nanotechnology—offers additional options to support more efficient and sustainable agricultural systems.

Nanomaterials exhibit size-dependent properties, such as a high surface-area-to-volume ratio, enhanced reactivity, tunable solubility, and distinctive mechanical, electrical, magnetic, optical, and thermal behaviour compared with their bulk [8,9,10,11]. These features have enabled applications in biomedicine, packaging, energy, water treatment, food-related technologies, and other sectors (Table S1) [12,13].

Within this broader field, polymer nanofibres obtained by electrospinning have become a particularly versatile platform [14,15]. Electrospinning is a scalable, relatively low-cost electrodeposition technology that uses electrostatic forces to draw continuous fibres from polymer solutions, suspensions, blends, or melts, typically with diameters in the 10 nm–1 µm range [16,17,18]. Nanofibrous structures have been explored in tissue engineering, drug delivery, filtration, antimicrobial materials, energy, environmental remediation, sensors, and agriculture (Table S2) [14,15]. Electrospraying, a related process, employs an electric field to generate micro- and nanoscale droplets that solidify into particles under controlled conditions [19,20,21]. Combining electrospinning and electrospraying enables simultaneous deposition of fibres and particles, broadening the range of organic and inorganic components, facilitating the encapsulation of bioactive compounds, and generating hybrid mats with tailored architectures and functions [22,23]. In agriculture, nanofibrous systems have been proposed for crop protection, controlled delivery of active substances, and sensing (Table S2).

In parallel, agro-industrial waste is increasingly recognised as a renewable feedstock for the production of value-added products [20]. Within the EU bioeconomy framework, agricultural residues are regarded as a resource rather than waste, provided they are channelled into value-added uses through a cascading scheme [24,25]. In this view, the extraction of high-value molecules and materials from agro-industrial streams should precede low-value options such as direct combustion. This approach is not marginal: agro-industries generate on the order of 1.3–2.1 billion tonnes of residues per year worldwide [26], and these materials are typically rich in lignocellulosic biomass (cellulose, hemicellulose, and lignin), proteins, minerals, and a broad array of secondary metabolites [27,28]. Agro-industrial waste is typically rich in functional organic matter components, inorganic constituents, and bioactive compounds (Table S3). Several products can be recovered from these residues (Table S4) and have already been exploited to recover biopolymers, biofuels, enzymes, and nutraceuticals as valuable materials, and, more recently, as precursors for bio-based nanomaterials and nanocomposites (Tables S3 and S4).

In this work, we focused specifically on lignin and polyphenols extracted from hazelnut shells and grape pomace, respectively, as agro-industrial waste to obtain eco-friendly functional ingredients.

Lignin is the second most abundant natural polymer after cellulose, accounting for roughly 5–35 wt% of plant biomass and 10–52 wt% of agro-industrial residues, depending on species and processing. It is a complex aromatic biopolymer, primarily located in the secondary cell walls of woody and vascular tissues (xylem and bark), built from p-coumaryl, coniferyl, and sinapyl alcohols linked via ether and carbon-carbon (C–C) bonds into a heterogeneous network [29]. Its composition reflects plant genetics, tissue type, and environmental conditions. Owing to its rigid, largely hydrophobic structure, lignin confers mechanical strength, facilitates water transport, and enhances resistance to microbial degradation. It also exhibits antioxidant, antifungal, antimicrobial, and UV-protective properties [29,30,31,32].

These characteristics have supported a growing range of applications in the food sector, biorefineries and biofuels, wood adhesives, biomedical materials, coatings, catalysts, surfactants, drug-delivery systems, packaging, and other functional materials [31,33,34,35,36,37,38]. The development of “nanolignin” has further expanded this portfolio: nanosized lignin often disperses more effectively, interacts more efficiently with polymer matrices, and can enhance mechanical, thermal, and barrier properties [27,39]. Electrospun lignin-containing fibres and nanocomposites have been proposed for drug delivery, filtration, energy devices, and biomedical applications [40,41,42,43], confirming that lignin can act both as a structural modifier and as a carrier or co-carrier for active substances [32,44,45,46,47]. In this study, lignin recovered from hazelnut shells was used as a bio-based functional additive in electrospun ± electrosprayed nanofibrous architectures.

**Figure 1:**
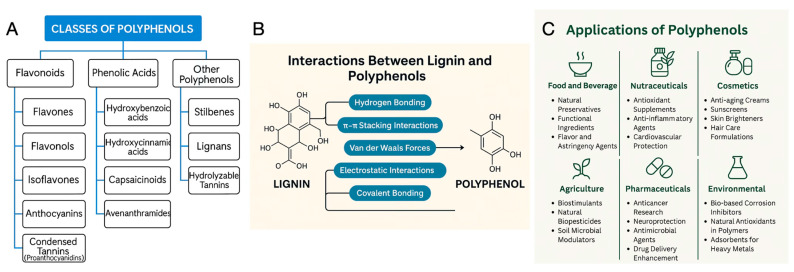
Schematic representation of (**A**) the classification of major polyphenol classes; (**B**) the main interaction mechanisms between polyphenols, lignin, and carrier polymers; and (**C**) their principal application domains. The scheme synthesises concepts reported in the literature [48,49,50].

Polyphenols are among the dominant secondary metabolites in plants and are widely distributed in fruits, vegetables, cereals, tea, coffee, and many other crops. More than 10,000 plant polyphenols have been identified [34]. They generally contain one or more hydroxylated aromatic rings and can occur as monomeric phenylpropanoids or as oligomeric and polymeric forms, such as proanthocyanidins [35,36,37]. Their biosynthesis involves the shikimate and phenylpropanoid pathways, which generate hydroxybenzoic and hydroxycinnamic acids and precursors for a broad range of compound classes. Based on structural features, polyphenols are commonly grouped into flavonoids (e.g., flavones, flavonols, isoflavones, anthocyanins, and condensed tannins), phenolic acids (hydroxybenzoic and hydroxycinnamic acids, capsaicinoids, and avenanthramides), and other polyphenols (stilbenes, lignans, and hydrolysable tannins) (Figure 1A) [34].

The number and position of hydroxyl and methoxy groups, and the presence of hydrophobic substituents (e.g., prenyl groups, alkyl chains), lead to a wide range of polarities, from highly hydrophilic molecules (e.g., gallic acid) to strongly hydrophobic ones (e.g., pterostilbene). Through these functional groups and their aromatic rings, polyphenols engage in hydrogen bonding, electrostatic interactions, hydrophobic interactions, and π–π stacking (Figure 1B) [48,49]. In plants, polyphenols contribute to defence against herbivores and pathogens through antimicrobial, antifungal, and deterrent activities, and they support tolerance to abiotic stresses such as drought, salinity, excess radiation, and temperature extremes by scavenging reactive oxygen species and helping preserve membrane integrity and DNA structure [50,51,52,53]. In soil–plant systems, they also influence nutrient cycles: they chelate micronutrients such as Fe, with consequences for P and other elements, retain Ca, Mg, and K on exchange sites, and slow litter and SOM turnover via protein complexation and direct inhibition of microbial growth or enzyme activities [53,54,55]. In addition, many polyphenols act as signals, mediating allelopathic interactions, legume–rhizobia recognition, and the stimulation or suppression of fungal germination and hyphal growth [52,53,56]. Anthocyanins and related flavonoids also contribute significantly to plant pigmentation and aroma, thereby influencing pollination and seed dispersal [51,52,57]. Through adsorption onto clays and oxides, polyphenols ultimately form organo–mineral complexes and contribute to the stabilised fraction of soil organic matter [54,58].

These structural features underpin their antioxidant, antimicrobial, anti-inflammatory, and anticancer activities and explain their use in food and beverages, nutraceuticals, biomedicine, cosmetics, agriculture, pharmaceuticals, and environmental technologies (Figure 1C). In addition, polyphenols have also been used in micro- to nanoengineered systems. Plant-derived polyphenols are used as green reducing and stabilising agents for metal and metal-oxide nanoparticles, as antioxidants and UV stabilisers in polymer nanocomposites, as antimicrobial and structuring components in biopolymer films, as building blocks for lignin- or tannin-based hydrogels, foams and coatings, and as functional interlayers in stratified nanostructures [59,60,61,62]. Polyphenols also form the second major group of value-added molecules that can be recovered from agro-industrial residues. In this study, polyphenols were extracted from agro-industrial residues such as grape pomace and incorporated into bio-based nanocarriers (nanofibres and bead-like depots) for agricultural delivery to support plant health.

To fabricate electrospun nanofabrics capable of encapsulating waste-derived polyphenols for controlled delivery to plants, polyhydroxybutyrate (PHB) and polycaprolactone (PCL) were selected as biodegradable carrier polymers compatible with both electrospinning and electrospraying.

PHB is a partially crystalline polyester that can be obtained synthetically or produced by microorganisms. It is biodegradable and biocompatible and exhibits thermoplastic properties comparable to those of conventional polyolefins such as polyethylene and polypropylene [63,64,65]. PHB has been proposed for biodegradable packaging, agricultural films, medical devices, and disposable items, thereby contributing to reduced plastic waste and circular-economy strategies [66,67,68].

PCL is a semi-crystalline, hydrophobic polyester with good mechanical strength and slow degradation [69,70]. Its properties and degradation behaviour can be tuned by incorporating natural fillers and additives, such as starch, cellulose, chitosan, lignin, gelatin, or silk [71], thereby supporting its use in tissue engineering and controlled-release systems [69]. In this work, PHB and PCL were used alone or in combination as carriers for lignin and polyphenols in electrospun ± electrosprayed scaffolds.

To provide a structured contextual framework supporting the rationale of the present study, Tables S1–S6 summarise key classes of nanomaterials, polymer nanofibres, agro-industrial waste components, value-added products derived from them, and selected physicochemical descriptors of polyphenols. These tables represent a structured literature-mapping effort based on peer-reviewed sources cited in the main reference list and are intended to consolidate dispersed background information relevant to the design of the investigated biohybrid systems.

This study integrates environmentally friendly components, i.e., PHB and PCL, lignin from hazelnut shells, and polyphenols from grape pomace, into electrospun ± electrosprayed biohybrid architectures designed as polyphenol-delivery systems for agricultural applications. The extracted substances and the resulting nanostructured fabrics were characterised to identify the physicochemical determinants of polyphenol release, with particular attention to the role of lignin content and distribution within the scaffolds.

Polyphenol release was evaluated at ambient temperature (~25 °C) (T_A_) and at 37 °C (T_37_) over 14-day soaking in phosphate buffer (Section 2.7). Ambient conditions reflect typical soil-application scenarios, whereas T_37_ was used as a stress temperature to assess the architectures’ thermal robustness. Such temperatures can occur at shallow soil depths in Mediterranean and other warm regions during hot periods, particularly in dry or sparsely vegetated soils, on dark or tilled surfaces, in plastic-mulched fields, on south-facing slopes, or on recently burned sites [72,73,74,75,76,77,78,79,80].

## 2. Materials and Methods

### 2.1. Materials

Poly[(R)-3-hydroxybutyric acid] (PHB, natural origin, cat. N. 363502), polycaprolactone (PCL, Mn = 45,000 g/mol, cat. N. 704105), acetic acid (≥99%), absolute ethanol (analytical grade), 2,2,2-trifluoroethanol (TFE), phosphate buffer (pH 7.4), methanol, ammonium dihydrogen phosphate, orthophosphoric acid, acetonitrile and phenolic standards (≥98% purity) were purchased from Sigma-Aldrich (Merck KGaA, Darmstadt, Germany). Syringe filters (0.22 μm, 33 mm) were from Sigma-Aldrich (Merck KGaA), Darmstadt, Germany.

Hazelnuts (*Corylus avellana* L.) were purchased from a supermarket in Southern Italy. Nuts were cracked with a nutcracker; the outer shells (HSs) were collected, milled to a powder using a stainless-steel blade mill, passed through a 250 μm sieve, aliquoted, and stored at −20 °C in hermetically sealed, polyethylene bags before further analysis.

Grapes (Primitivo di Gioia del Colle, Azienda Agricola F.lli Rossi Soc. Agr. srl—Centovignali, Bari, Italy) were harvested at 24 °Brix, destemmed, and crushed. The must was supplemented, per 100 kg of grapes, with 4 g of potassium metabisulfite (K_2_S_2_O_5_), 30 g of dry yeast (Zymaflore FX10, Laffort, Bordeaux, France), and 20 g of diammonium phosphate [(NH_4_)_2_HPO_4_] (D&C Wine S.p.A., Faenza, Italy). Alcoholic fermentation was conducted at 22 °C for 15 days with two daily manual punch-downs. At the end of fermentation, pomace was separated and pressed (Torchietto “Premi Tutto” ALU20 Medio, Polsinelli Enologia, Isola del Liri, Italy) and stored at 4 °C until extraction.

#### 2.1.1. Lignin Extraction

**Figure 2:**
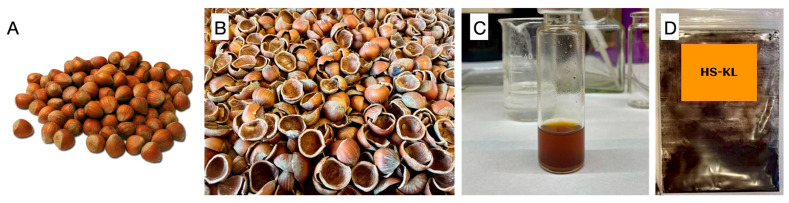
Steps of hazelnut shell processing from harvesting (**A**) to waste formation upon outer shell (HS) cracking (**B**), to lignin extraction from HS (HS-KL: Hazelnut-shell Klason lignin) (**C**), and finally to its powder upon vacuum drying (**D**).

Lignin was obtained as acid-insoluble lignin by the Klason method from hazelnut shell powder, according to TAPPI T 222 om-02 [81], with minor modifications (hereafter HS-KL) [39]. Briefly, 5 g of HS powder were treated with 150 mL of 72% (*v*/*v*) H_2_SO_4_ (solid-to-liquid ratio 1:30 *w*/*v*) at room temperature for 16 h. The mixture was diluted to 3% (*v*/*v*) with deionised water and heated at 105 °C under stirring (300 rpm) for 4 h. The suspension was vacuum-filtered on Whatman N°2 paper to collect the acid-insoluble lignin (hereafter HS-KL, Hazelnut-shell Klason lignin), which was repeatedly washed with distilled water (≥10 × 10 min washing cycles) until neutral pH was reached, then dried under vacuum at 80 °C (IKA HB 10 basic rotary evaporator, IKA-Werke GmbH & Co. KG, Staufen, Germany) and stored at 7 °C in dry conditions [82]. Extraction yield was calculated gravimetrically on a dry-weight basis relative to the mass of the initial HS powder (Figure 2).

#### 2.1.2. Polyphenol Extraction

Grape pomace was frozen in liquid nitrogen and ground using an IKA analytical batch mill (IKA-Werke GmbH & Co. KG, Staufen, Germany). Four grams of powder were extracted with 40 mL of methanol:water (80:20 *v*/*v*) in a low-temperature ultrasonic bath for 20 min. Samples were centrifuged at 11,200× *g* for 15 min at 4 °C, and the supernatant was used for polyphenol analysis.

The grape-pomace polyphenols (GP-PPs) present in the extract were identified and quantified by HPLC, following the method of Ritchey and Waterhouse (1999) [83]. A 10 mL aliquot was filtered through 33 mm-diameter 0.22 μm syringe filters (Sigma-Aldrich, Italy), diluted 1:20 (*v*/*v*) with Milli-Q water, and 1 mL was transferred to 2 mL amber vials. Analyses were performed using a Dionex HPLC system (P680 pump, manual injector with 20 μL loop, TCC-100 oven, PDA-100 detector, Chromeleon v.6.50; Thermo Fisher Scientific, Waltham, MA, USA). Separation was carried out on a C18 column (Dionex Acclaim^®^ 120 C18, 5 μm, 4.6 × 250 mm).

The mobile phase consisted of: solvent A, 50 mM ammonium dihydrogen phosphate (pH 2.8, orthophosphoric acid); solvent B, 20% A/80% acetonitrile; and solvent C, 0.2 M orthophosphoric acid (pH 1.5, NaOH). Flow rate was 0.5 mL/min at 40 °C. Quantification was based on a 5-point calibration (0.1–200 mg/L) using phenolic standards.

### 2.2. Electrospinning and Electrospraying Solutions

Nanostructured frameworks were prepared by combining biodegradable polymers (PHB, PCL) with lignin and polyphenols extracted from agro-industrial waste.

PHB and PCL stock solutions were prepared by dissolving PHB (228 mg mL^−1^) and PCL (266.67 mg mL^−1^) in 2,2,2-trifluoroethanol (TFE). TFE was selected because it dissolves both polymers and promotes bead formation during PCL electrospinning, thereby enabling fibre-and-bead architectures.

Hazelnut-shell Klason lignin (HS-KL) was dissolved in acetic acid (80 mg mL^−1^). Polyphenols from grape pomace (GP-PPs) were diluted in methanol:water (80:20 *v*/*v*) to 100 mg mL^−1^. Seven formulations were prepared by mixing PHB, PCL, HS-KL, and GP-PP in different ratios (Table 1). For each formulation, the electrospinning ± electrospraying process was continued until the syringe contents were fully discharged.

All solutions were sonicated using a probe sonicator (6000 J mL^−1^, Vibra Cell VCX 400, Sonics and Materials Inc., Newtown, CT, USA), vortexed, and magnetically stirred at room temperature until complete homogenisation.

### 2.3. Electrospinning/Electrospraying of Nanostructured Frameworks

Four nanostructured fibrous fabrics were produced by combining the seven formulations in different ways and, in selected cases, coupling electrospinning and electrospraying in a Fluidnatek^®^ LE-50 system (Bioinicia, Paterna, Spain), under the conditions reported in Table 1. Table 1 also summarises the mass ratios of the various components in each final scaffold.

### 2.4. Morphological Characterisation

#### 2.4.1. Stereomicroscopy and Optical Microscopy

Scaffold fragments were mounted on thin SiO_2_ wafers. Stereomicroscopy was performed with an Ivesta 3 Greenough stereo microscope with integrated camera (Leica Microsystems GmbH, Wetzlar, Germany). Optical microscopy was performed using a DM2700 M microscope equipped with a K5C Colour CMOS camera (Leica Microsystems GmbH, Wetzlar, Germany). These observations provided an overview of the surface texture, fibre network, and the presence of dark particles before and after 14-day soaking in 0.11 M phosphate buffer (pH 7.4) at 37 °C.

#### 2.4.2. Scanning Electron Microscopy and Image Analysis

Morphology was further analysed by field-emission scanning electron microscopy (FE-SEM) using a Tescan MAGNA GMU (Tescan, Brno, Czechia) equipped with an AztecLive EDS system with Ultim Max 65 detector (Oxford Instruments, Abingdon, UK). Samples were electrospun directly onto silicon wafers with native SiO_2_, mounted on aluminium stubs with conductive carbon tabs, and sputter-coated with ~5 nm Au.

SEM micrographs were acquired in secondary-electron mode at magnifications of 2k×, 5k×, 10k×, 15k×, and 20k×, with accelerating voltages between 2 and 20 kV, beam current of 30 pA, and field-of-view between ~17 and 70 μm. Several images per sample were collected to assess homogeneity and capture key features (fibre diameter, bead formation, surface roughness, globular/embedded structures).

Average PHB and PCL fibre diameters were measured on comparable micrographs using the DiameterJ v.1-018 plugin in ImageJ 1.51k (≥102 measurements per scaffold from three different pieces). For matrices where unexpected PCL nanofibres formed during intended electrospraying, these were excluded from fibre-diameter statistics, as the design rationale was to compare planned morphologies and their effect on lignin/polyphenol behaviour.

Particle area and roundness were measured on PHB and PCL particles generated by electrospraying (PHB and PCL) or electrospinning (PCL), based on SEM images analysed with ImageJ 1.51k (≥51 measurements per scaffold from three pieces). Roundness, as calculated by the software, is a dimensionless shape descriptor that quantifies how closely a particle resembles an ideal circle. Values approaching 1 correspond to nearly spherical particles, whereas progressively lower values indicate increasingly elongated, irregular, or deformed shapes. This parameter was employed to monitor potential morphology changes, such as deformation or surface erosion, before and after 14-day soaking in 0.11 M phosphate buffer (pH 7.4), regardless of particle size or origin (electrosprayed vs. electrospun).

### 2.5. Interactions of the Nanohybrid Scaffolds with Water

#### 2.5.1. Water Contact Angle (WCA) Measurements

Dynamic WCA was measured with a custom-built setup equipped with a Supereyes B011 5 MP digital USB microscope (Supereyes, Shenzhen, China). A 7 μL droplet of distilled water was deposited on the scaffold surface using a calibrated micropipette. Droplet profiles were recorded at 0, 30, 60, 90, 120, 180, 240, 300, 450, and 600 s. Contact angles (θ) and droplet volumes were obtained by drop-shape analysis (axisymmetric drop shape analysis, ADSA) using the Drop Analysis LB_ADSA plugin in ImageJ.

#### 2.5.2. Water Absorption/Infiltration

Water uptake was expressed as the percentage change in water droplet volume (WDV) over time relative to the initial 7 μL (100%). Volumes at each time point were extracted from the same image series used for WCA by ADSA analysis, enabling assessment of evaporation versus absorption/infiltration into the porous scaffolds.

### 2.6. Spectroscopic Characterisation

#### 2.6.1. UV–Vis Spectroscopy

UV–Vis spectra of HS-KL and GP-PP solutions were acquired between 185 and 700 nm using a UV-2600 spectrophotometer (Shimadzu, Kyoto, Japan). Polyphenols were measured in methanol:water (80:20 *v*/*v*) extracts (1 mg mL^−1^), which were subsequently dissolved in 0.11 M phosphate buffer (pH 7.4) at 37 °C to maximise solubility. HS-KL was dispersed in phosphate buffer and analysed by UV–Vis spectroscopy under the same conditions.

UV–Vis spectroscopy was also used to monitor GP-PP release by measuring the absorbance of phosphate buffer solutions in which the scaffolds were soaked for 14 days at either ~25 °C or 37 °C (Section 2.7).

#### 2.6.2. Fourier Transform Infrared Spectroscopy (FTIR-ATR)

Fourier transform infrared (FTIR) spectra of lignin were recorded using a Spectrum 3 Tri-Range MIR/NIR/FIR spectrometer (PerkinElmer, Waltham, MA, USA) equipped with a Universal ATR diamond crystal. Spectra were collected in the 4000–650 cm^−1^ range at 4 cm^−1^ resolution, with 16 scans per sample at room temperature. Characteristic lignin bands were used to confirm the presence of typical functional groups in the extracted Klason lignin.

### 2.7. Polyphenol Release from the Nanostructured Scaffolds

Grape-pomace polyphenol (GP-PP) release was assessed by UV–Vis analysis of buffer solutions in which the nanostructured fabrics were immersed. Diffusion-driven release is temperature-dependent through the diffusion coefficient and polymer mobility; therefore, experiments were performed at two temperatures: ambient (~25 °C, T_A_) and 37 °C (T_37_), which was used as the stress temperature.

Scaffold strips (1 cm × 5 cm; 5 cm^2^) were immersed in 5 mL of 0.11 M phosphate buffer (pH 7.4). For each scaffold and temperature, the incubation medium was collected in full at each sampling time (daily over 366 h-14 days, with an additional sampling point at 6 h on day 1). An aliquot of the collected solution was used for UV–Vis analysis, and the scaffold was subsequently incubated in fresh buffer (5 mL) for the next time interval.

The 0.11 M phosphate buffer (pH 7.4) was chosen to provide a stable aqueous medium with a pH representative of Mediterranean agricultural soils, which typically range from slightly acidic to moderately alkaline with mean values around 7.4 (4.3–8.6) [84,85]. This avoided pH-driven artefacts in polyphenol spectra and ensured comparability across matrices.

UV–VIS spectra of the release media exhibited two main polyphenol-related peaks (λ_1_ ≈ 208 nm and λ_2_ ≈ 280 nm) and a shoulder around 320–330 nm (Section 3.1.3). Peak areas were integrated using the instrument’s software (UV Probe Ver. 2.50). For quantitative analysis, the shoulder contribution was merged with the λ_2_ peak. Polyphenol release was expressed as arbitrary units.

For each scaffold and time point, the total polyphenol-related area (*A_total_*) was defined as

*A*_*total*_ = *A*_1_ + *A*_2_, (1)

where *A*_1_ is the integrated area at *λ*_1_ (≈208 nm), and *A*_2_ is the integrated area at λ_2_ plus shoulder (≈280–330 nm). These areas reflected the combined contributions of multiple polyphenolic species in the extract and thus represented the overall polyphenol content rather than those of individual compounds (Supplementary Materials, §S4).

Normalised polyphenol release (PP_norm_) was then calculated as

(2) PPnormt=Atotal(t)mPP,

where *m_PP_* is the estimated theoretical mass of GG-PP loaded in the tested strip (based on initial formulation). This normalisation allowed comparison across scaffolds with different initial GP-PP loadings. Based on the GP-PP release measurements obtained from the procedure above, the GP-PP release profile over time performed at T_A_ and T_37_ was quantified, and various phases were identified by analogy with controlled-release fertiliser (CRF) descriptors: *burst*, *mid*, *late tail*, *total* released area, *late fraction*, and t_50_. Specifically, the *burst* = the period including the first peak; *mid* = includes the second peak; *late tail* = includes the third peak plus the terminal shoulder, if present; *total* = the total released polyphenols over the entire period of measurements; *late fraction* = *late tail* area/*total* area, i.e., the proportion of the total that occurs in that same late window; t_50_ = time at which the cumulative release area reaches 50% (linear interpolation between the timepoints). Because release profiles were multimodal, phase areas were quantified primarily by a peak-centred approach. Peak domains were then delimited by inter-peak minima, yielding *burst* (first peak), *mid* (second peak), and *late tail* (third peak + terminal shoulder) contributions. A fixed-window integration was used only as a secondary sensitivity analysis for cross-condition comparability among the matrices and the temperatures tested: *burst* (0–78 h), *mid* (78–192 h), and *late tail* (>192 h).

#### 2.7.1. Daily Release Normalisation and Comparison

To compare scaffolds on a daily basis within the same matrix type, the daily polyphenol release of each scaffold was first normalised to the total amount of polyphenols released over the entire experimental period (14 days) by that specific matrix, which was set to 100%. The relative daily contribution of each scaffold *i* was then calculated as a percentage of the matrix’s total daily release.

(3) PPr−release/day(i)%=Ai(day)∑j=1nAj(day), 

where $A_{i}\left(day\right)$ is the daily released polyphenol signal (e.g., UV–Vis area) of scaffold *i*, expressed as a fraction of the total amount released over 14 days by the same matrix (100%), and the denominator is the sum of the daily released fractions over all *n* scaffolds belonging to that matrix. This normalisation enabled a scaffold-by-scaffold comparison within each matrix type, independent of differences in absolute release among the four matrix formulations.

#### 2.7.2. Polyphenol Release Rate Trend Analysis

Cumulative normalised release curves were obtained by summing PP_norm_ over time for each scaffold. To provide a first-order approximation of release kinetics and facilitate comparison, linear regressions were fitted to the cumulative data as

y = ax + b,(4)

where *y* is the cumulative normalised release (sum of peak areas divided by *m_PP_*), *x* is time (h), *a* is the apparent release rate (area units·h^−1^), and *b* is the intercept. The coefficient of determination (R^2^) (OriginPro 2016, OriginLab) was used to assess goodness of fit over the whole 14-day period (~340 h) and to compare overall release trends across scaffolds and temperatures.

## 3. Results and Discussion

In this study, we aimed to develop environmentally friendly products for sustainable agriculture applications. To achieve this goal, we employed various low-impact components from different sources. In typical drug delivery systems that act on organisms, a carrier architecture encases bioactive substances that must be released outward to perform their functions. In detail, we utilised polyhydroxybutyrate (PHB) and polycaprolactone (PCL) as biodegradable carrier polymers to create various architectures using electrospinning ± electrospraying as nanotechnological techniques. Then, we extracted lignin and polyphenols from agro-industrial waste as valuable compounds to engineer bio-based, controlled-release polyphenol products that support plant growth.

### 3.1. Extraction and Characterisation of Valuable Bio-Based Compounds from Agro-Industrial Waste

#### 3.1.1. Yield and Recovery of Lignin

**Figure 3:**
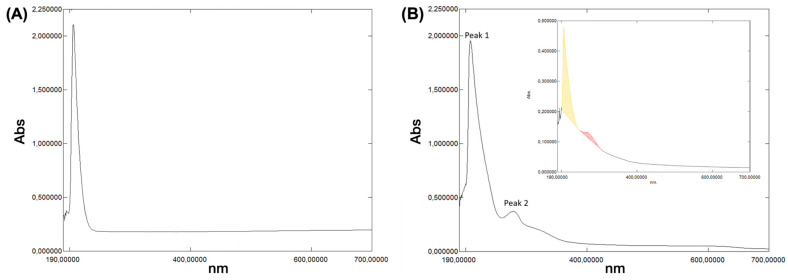
UV–Vis absorbance spectra, in the range 195 nm–700 nm, of lignin extracted from hazelnut shells (**A**). (**B**) UV–Vis absorbance spectra, in the range 195 nm–700 nm, of the polyphenolic extract from grape pomace, depicting two characteristic peaks: Peak 1 (207.5 nm) and Peak 2 (278.5 nm). The inset in B shows an example of how the peak areas were calculated for quantitative analysis; the yellow shaded region corresponds to the integrated area of Peak 1, while the red shaded region corresponds to the integrated area of Peak 2.

Lignin is a key component of agro-industrial waste. We employed Klason lignin from hazelnut shells as a reinforcing and potentially release-modulating co-component, either embedded or co-deposited with polyphenols in nanocomposite PHB ± PCL architectures, to modulate mechanical stability and hydration and ultimately generate polyphenol-loaded fabrics with controlled-release properties. Klason lignin (acid-insoluble fraction) (HS-KL) was extracted from milled hazelnut shell powder according to the two-step acid hydrolysis protocol described in Section 2.1.1. The procedure was readily implemented under laboratory conditions and yielded reproducible results. On a dry-weight basis, the extracted HS-KL was ~48.9 wt% of the starting hazelnut shell material, confirming that this residue was lignin-rich and suitable as a feedstock for lignin recovery within a circular bioeconomy context. Figure 3A shows the UV–Vis absorbance spectrum of the extracted HS-KL.

#### 3.1.2. Yield and Recovery of Polyphenols

Polyphenols were extracted from grape pomace (GP-PP) following the method reported in Section 2.1.2. HPLC–PDA analysis of the extract revealed five main polyphenol classes, including anthocyanins, flavan-3-ols, flavonols, phenolic acids, and stilbenes (Table 2) (Figure S1). Polyphenols were combined with carrier polymers and lignin to tailor the architectures and, consequently, the polyphenol release behaviour. Based on the composition of the extract, polyphenols were predominantly hydrophilic, with only a minor contribution from more hydrophobic species (Table S5). The polyphenol polarity profile is relevant to both interactions with PHB and PCL during electrodeposition and to the subsequent release behaviour in aqueous buffer. To achieve this goal, polyphenols were dispersed either within the electrodeposited fibres and/or in bead-like depots comprising the scaffolds.

#### 3.1.3. Valuable Spectroscopic Characterisation of the Extracted Substances

##### UV–Vis Characterisation of the Grape-Pomace Extract

The UV–Vis spectrum of the methanol:water (80:20, *v*/*v*) grape-pomace extract displayed two main absorption regions (Figure 3B): a strong band at ~208 nm (Peak 1) and a second band at ~280 nm (Peak 2) with a shoulder around 320–380 nm (Figure 3B). The first peak is characteristic of intense and high-energy far-UV π–π* transitions in aromatic rings (“E/B” band), while the second band is consistent with lower-energy π–π* transitions (Band II), often described as “benzenoid/benzoyl system” transitions in flavonoids and due to hydroxyl and carbonyl groups within aromatic structures typical of phenolic acids (like gallic acid) and flavan-3-ols. Moreover, the slight 320–380 nm shoulder is typical of π–π* transitions (Band I) in flavonols and flavones. Typically, plant extracts are composed of a multitude of different polyphenols (flavonoids, phenolic acids, stilbenes, hydrolysable tannins, lignans, etc.) (Figures S2 and S3D). In addition, solvents used for the UV–Vis measurements and pH further modulate peak position and intensity in UV–Vis absorbance spectra (§S4) [44,86]: acidic polyphenols dissolved in 0.11 M phosphate buffer (pH 7.4) at ~25 °C exhibit reduced absorbance and slightly shifted maxima relative to more acidic conditions, consistent with reported bathochromic and hypochromic trends [39,40,41,42,43,44,45,86,87,88] (Figure S3A,B).

Furthermore, plant extracts contain not only several polyphenol classes but also other cell-derived solutes. Hence, the resulting UV–Vis absorbance spectra will represent the superposition of multiple plots. A simple spectral reconstruction is reported in Figure S3, combining typical spectra of free polyphenols, proanthocyanidins, tannin–protein complexes, and minor soluble proteins, and reproducing the main features of the measured spectrum (Figure S3C) [39,40,41,88]. The intense 208 nm peak can therefore be interpreted as arising from overlapping contributions of free and protein-bound polyphenols and condensed tannins and is typically much higher than the others [89], whereas the 280 nm band is mainly associated with phenolic acids/flavan-3-ols, tannic acid, anthocyanins, and proanthocyanidins [41,46,87] (Figure S3C).

Direct HPLC–PDA detection at 280 nm confirmed the presence of gallic acid, procyanidin dimers, (+)-catechin, and 4-hydroxybenzoic acid, additional to other polyphenol compounds (Table 2, Figure S1), which is consistent with both the simulated composite UV-Vis spectrum (Figure S3C) and the wine and pomace phenolic profiles (Figure S3D) [42,43].

##### UV–Vis Characterisation of Klason Lignin from Hazelnut Shells

The UV–Vis spectrum of HS-KL exhibited a strong absorption band at ~207 nm, with a much weaker, broader feature centred at ~480 nm (Figure 3A). The deep-UV band reflects π–π* transitions in the aromatic and conjugated structures typical of lignin, while the low-intensity visible band is commonly ascribed to minor chromophores or extended conjugation domains.

##### FTIR-ATR Characterisation of Klason Lignin from Hazelnut Shells

**Figure 4:**
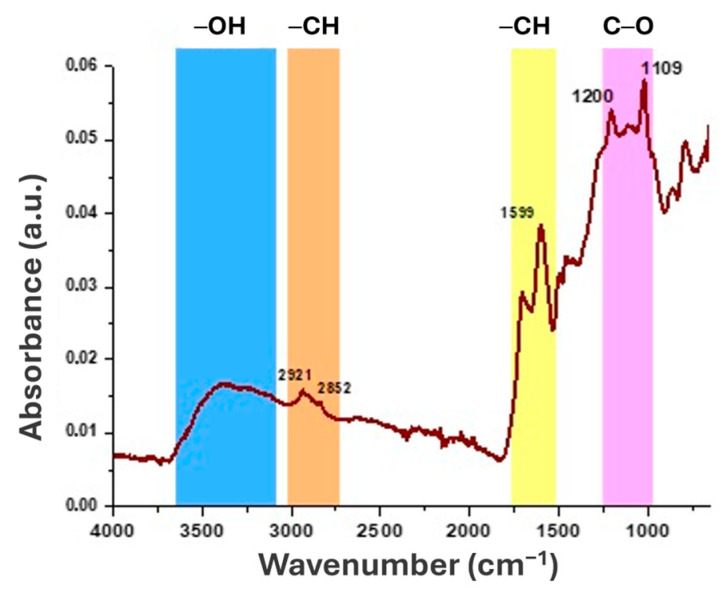
FTIR-ATR spectrum of purified Klason lignin (acid-insoluble fraction) from hazelnut shells (HS-KL).

The FTIR-ATR spectrum of HS-KL displayed the expected signatures of lignin macromolecules. In Figure 4, a broad band around 3300 cm^−1^ corresponds to O-H stretching vibrations of phenolic and aliphatic hydroxyl groups. Two weaker bands at approximately 2921 and 2852 cm^−1^ can be assigned to C-H stretching in aromatic and aliphatic moieties [47]. In the fingerprint region, intense bands between 1600 and 1100 cm^−1^ reflect the lignin aromatic backbone. The peaks at ~1600 and ~1500 cm^−1^ are associated with aromatic skeletal vibrations, whereas the band at ~1450 cm^−1^ is related to methoxy groups in guaiacyl and syringyl units, the main lignin monomer types [82]. Furthermore, the peak at 1200 cm^−1^ is attributed to vibrations of methoxy groups and to C-O stretching and deformation in secondary alcohols and aliphatic ethers. Finally, a distinct signal at ~1109 cm^−1^ may indicate partial incorporation of sulfate groups into lignin molecular structures during the concentrated H_2_SO_4_ treatment [82], consistent with the Klason method extraction protocol used in this study (Section 2.1.1).

### 3.2. Structural Characterisation of Biohybrid Nanocomposites

#### 3.2.1. Design Logic and Matrix Composition

**Figure 5:**
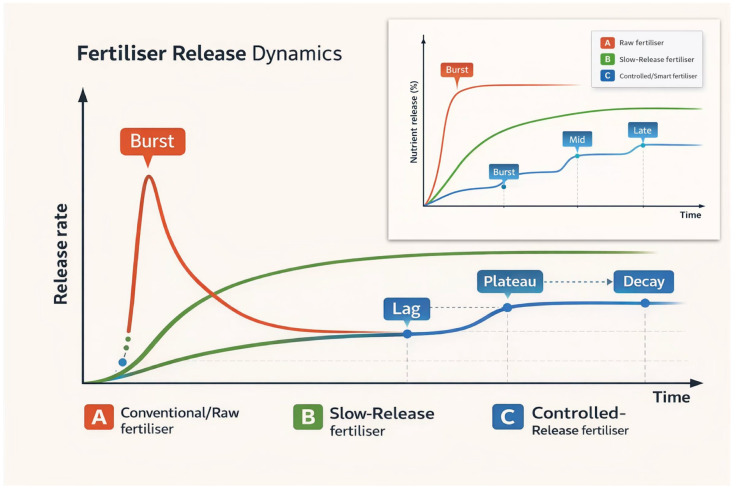
Schematic of nutrient release dynamics for different types of fertilisers: conventional/raw fertilisers; slow-release fertilisers (SRFs); controlled-release fertilisers (CRFs). Nutrient release rate (A); Cumulative nutrient release (%).

The overarching goal of this work was to construct biohybrid nanocomposite matrices that, in functional terms, behave like multimodal controlled-release formulations: a limited initial *burst* followed by *mid* and *late* phases that can be tuned through material selection and architecture (Figure S4) [86,90,91,92,93]. Rather than encapsulating mineral nutrients, the scaffolds were designed to deliver a polyphenol-rich grape-pomace extract as a bioactive cargo for plants. The design therefore mirrors the logic of controlled-release fertilisers (CRFs), more than slow-release fertilisers (SRFs), in which nutrient release is governed by coating composition, layer structure, and environmental conditions (Figure 5) [94,95,96,97], but is implemented here using biodegradable polyesters and agro-waste-derived additives.

To this end, four matrices (MatA-D) were engineered by combining two carrier polymers (PHB and PCL), Klason lignin (acid-insoluble fraction) from hazelnut shells (HS-KL), and polyphenols from a grape-pomace extract (GP-PP), using electrospinning and electrospraying (Table 1). The matrices differ systematically in the following:

- *Polymer phase organisation:* Single-polymer fibrous networks (PHB) versus multiphase fibrous architectures obtained by the co-deposition of PHB and PCL from separate electrospinning nozzles;
- *Localisation of HS-KL:* Confined to PHB fibres vs. distributed across both PHB and PCL phases vs. absent;
- *Localisation of GP-PP:* Restricted to bead-like depots vs. distributed between fibrous networks and bead-like structures;
- *Architecture:* Fibrous networks alone or combined with bead-type depots of different sizes and loading.

All matrices were tested at ambient temperature (T_A_) and at 37 °C (T_37_) as well as upon short- and long-term exposure to aqueous solutions. In brief (see Table 1 for the full compositions),

- ***MatA*** is a PHB-only scaffold. HS-KL is confined to electrospun PHB fibres, whereas GP-PPs are loaded into PHB particles generated by electrospraying (co-deposition). Lignin and polyphenols do not co-exist in the same domains.
- ***MatB*** is a PHB/PCL composite. PHB+HS-KL fibres form the structural network, whereas large PCL+GP-PP particles produced by electrospraying serve as the primary depots (co-deposition). Again, HS-KL and GP-PP reside in different polymer phases.
- ***MatC*** contains both PHB and PCL fibres and PCL bead-on-string segments (co-deposited) in which HS-KL and GP-PP are co-located. Both polymers, therefore, act as carriers for HS-KL-GP-PP microdomains distributed across fibres and beads.
- ***MatD*** has the same PHB/PCL architecture as MatC but contains GP-PP only, with no HS-KL. It provides a reference system in which polyphenols function solely as cargo and plasticisers, without lignin-mediated metering.

These four matrices thus span three key design axes: (i) PHB vs. PCL as carrier phases; (ii) presence/absence and placement of lignin; and (iii) segregation vs. co-location of HS-KLs and GP-PPs. This structural diversity underpins the different wetting, swelling, and release behaviours described in the subsequent sections.

#### 3.2.2. Polymer Phase and MAF/RAF Microstructure

PHB and PCL are both semicrystalline polyesters, but with distinct thermal windows and microstructures. PHB crystallises readily and has a higher glass transition temperature, whereas PCL is more rubbery at ambient conditions. When electrospun or electrosprayed, both polymers develop the classical three-phase microstructure of semicrystalline polymers: (i) crystalline lamellae, (ii) a mobile amorphous fraction (MAF), and (iii) a rigid amorphous fraction (RAF) at crystal interfaces [90,91,92,93,98]. MAF provides the main pathways for water ingress and solute diffusion; RAF is less mobile and behaves as an interfacial “shell” around crystals.

Although detailed crystallinity values were not measured here, the combination of polymer identity, fibre vs. bead morphology, and processing route suggests a qualitative hierarchy, PHB fibres > PHB beads > PCL fibres > PCL beads, in terms of overall crystallinity/rigidity (more crystals + RAF, less MAF). Coarser PHB fibres tend, therefore, to act as slower, more gated diffusion pathways, whereas PCL beads—especially the larger ones in MatB and the bead-on-string elements in MatC/D—contain the most accessible and continuous MAF and are expected to act as high-capacity depots for polyphenol release.

In practical terms, this means that (i) PHB-rich regions should contribute to structural stability and late-phase gating, and (ii) PCL-rich regions, particularly beads, should dominate the *mid* and *late* portions of the release curves, provided they do not densify excessively during ageing.

#### 3.2.3. Lignin as a Structural and Interfacial Modifier

Within this semicrystalline framework, Klason lignin (HS-KL) plays a dual role. Structurally, its phenolic–aromatic framework can interact with PHB and PCL through hydrogen bonding and π–π interactions, influencing chain packing and crystallisation. Depending on concentration and local environment, HS-KL can: (i) act as a nucleating agent, promoting formation of smaller crystals and increasing the RAF; (ii) or partially disrupt packing due to its bulky, irregular structure, thereby increasing the continuity of MAF [91,92].

In either case, HS-KL-containing regions tend to become mechanically stiffer and less prone to uncontrolled swelling, as observed later for fibre swelling at T_37_. This contributes to the greater morphological stability of matrices that contain lignin in their fibrous framework (MatA-C) compared with the HS-KL-free system (MatD).

At the interface, lignin provides a dense distribution of hydroxyl and aromatic sites that can bind polyphenols via hydrogen bonding and π–π stacking (Figure 1B). When HS-KL and GP-PP are co-located in the same polymer phase (MatC), these interactions (i) help to retain GP-PP within specific microdomains [99,100,101,102], (ii) reduce their ability to plasticise the polyester matrix, and (iii) generate “interfacial depots” where diffusion is metered by reversible HS-KL–GP-PP binding.

When HS-KL and GP-PP are spatially separated (MatA and MatB), lignin still improves fibre stability and wettability but cannot directly meter depots, so its effect on release is more indirect.

#### 3.2.4. Polyphenol Mixture and Expected Partitioning

The grape-pomace extract used here contains several polyphenol classes (Table 2), which span a broad polarity range (Table S5): (i) small, highly hydrophilic molecules such as gallic and 4-hydroxybenzoic acids, (ii) anthocyanins, generally hydrophilic and cationic/zwitterionic near neutral pH, (iii) intermediate-polarity flavonols (e.g., catechin-derived structures), and (iv) more hydrophobic species, including stilbene-type molecules.

On the basis of their polarity and aromaticity, a simplified partitioning picture can be drawn: (i) hydrophilic acids and anthocyanins preferentially reside in hydrated MAF regions and near HS-KL-rich interfaces; (ii) flavonols can bridge between HS-KL-rich domains and more hydrophobic segments of PHB/PCL; and (iii) hydrophobic stilbene-like species tend to partition into less hydrated, more hydrophobic MAF (PCL > PHB), again interacting strongly with lignin where present.

Accordingly, in matrices where HS-KL and GP-PP are co-located (MatC), one expects a hierarchy of retention: hydrophilic acids and anthocyanins form relatively labile, early-releasing complexes; flavonols and stilbenes are held more strongly and contribute disproportionately to the *mid* and *late* phases. In HS-KL-free matrices (MatD), the same compounds act mainly as plasticisers: they ease chain mobility and water uptake but are less effectively “held back” by specific binding, so structural changes can outpace controlled metering.

### 3.3. Morphological Characterisation of the Bio-Based Nanohybrids

#### 3.3.1. Stereomicroscopy

**Figure 6:**
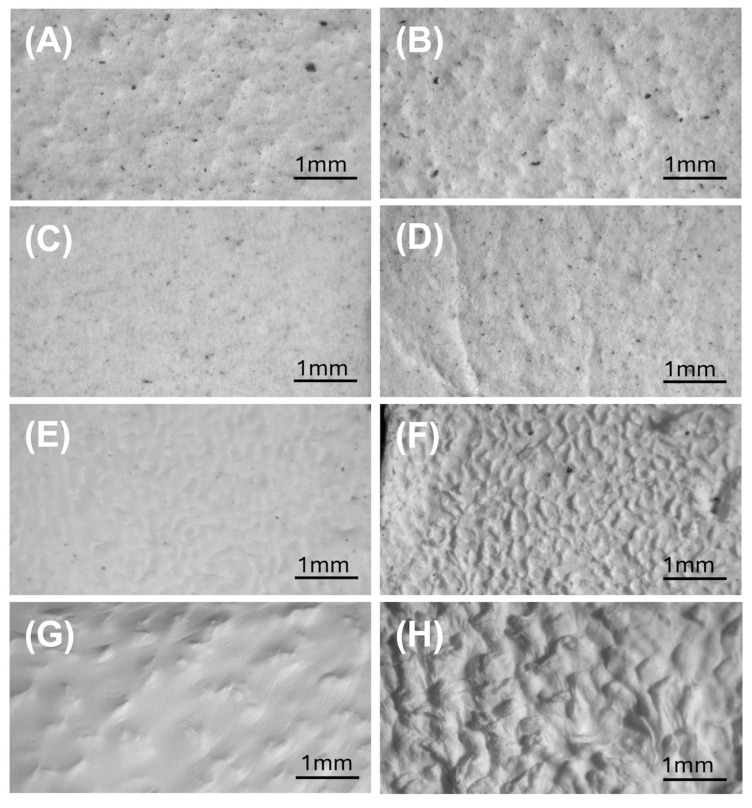
Comparison of nanostructured scaffolds MatA, MatB, MatC, and MatD before (**A**,**C**,**E**,**G**) and after (**B**,**D**,**F**,**H**) 14-day soaking in the 0.11 M phosphate buffer (pH 7.4) at 37 °C, as observed by stereomicroscopy.

Bulleted stereomicroscopy provided an initial low-magnification overview of scaffold architecture before and after 14-day soaking in 0.11 M phosphate buffer (pH 7.4) at T_37_, i.e., under the same conditions used for the release tests. All mats appeared macroscopically continuous, but surface texture and graininess differed (Figure 6).

Before 14-day soaking in 0.11 M phosphate (pH 7.4), MatA and MatB (Figure 6A,C) showed a relatively rough, granular surface with numerous dark particles, whereas Mats C and D (Figure 6E,G) were smoother, with MatC often displaying a convoluted, “brain-like” topography and MatD a more silky, weakly wrinkled one. Immersion in 0.11 M phosphate buffer (pH 7.4) for 14 days generally increased surface roughness, particularly in Mats B–D, where depressions and ridges became more pronounced (Figure 6D,F,H). Given that all scaffolds are nanofibrous, this increased roughness is consistent with partial fibre swelling, collapse, and local surface fusion. Dark grains were abundant and broadly distributed in Mats A–C, but essentially absent in MatD. Microscopical observations at the millimetre scale do not show an evident decrease in the surface density of dark lignin-rich domains after 14 days of immersion at 37 °C, except for MatA.

#### 3.3.2. Optical Microscopy

**Figure 7:**
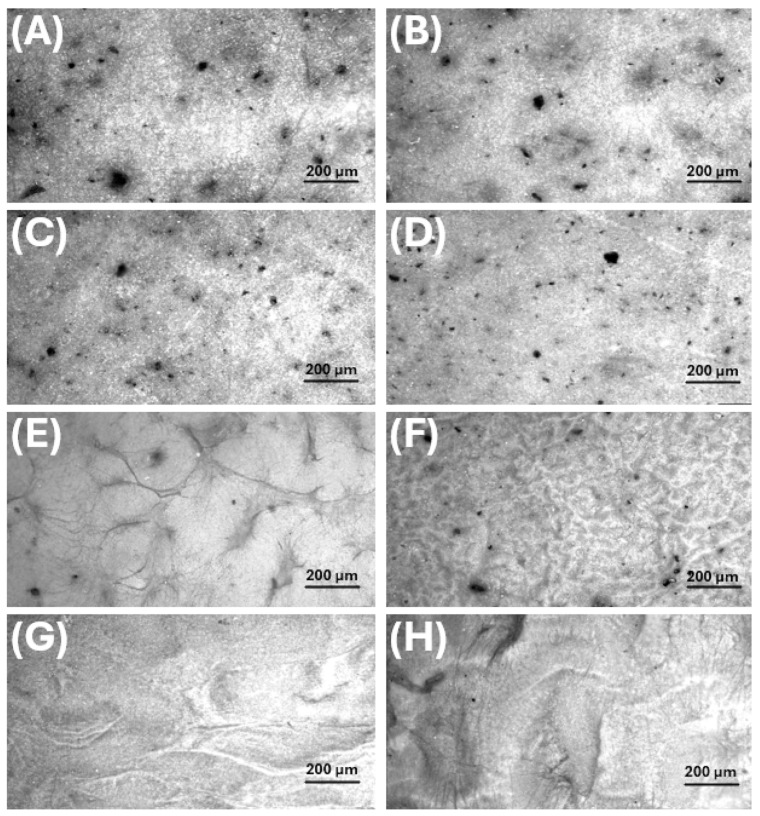
Comparison of nanostructured scaffolds MatA, MatB, MatC, and MatD before (**A**,**C**,**E**,**G**) and after (**B**,**D**,**F**,**H**) 14-day soaking in the 0.11 M phosphate buffer (pH 7.4) at 37 °C, as observed by optical microscopy.

Optical microscopy, at higher magnification, emphasised the nanofibrous contribution to the architecture and refined the stereomicroscopic observations (Figure 7).

In Mats A and B (Figure 7A,C), a dense PHB fibre network was visible, with fibres of different diameters interlaced with darker, more compact domains corresponding to the bead-like depots. The presence and distribution of dark lignin-rich grains observed by stereomicroscopy were also confirmed after 14-day soaking in 0.11 M phosphate buffer (pH 7.4) (Figure 7B,D).

In MatC, the unsoaked scaffold appeared as a clear, finely entangled fibrous web. After immersion, its surface acquired a characteristic convoluted, “brain-like” appearance, consistent with limited fibre coalescence and local reorganisation at the surface, without gross collapse of the fabric (Figure 7E,F). In MatD, the lignin-free network exhibited broader wrinkles even before 14-day soaking; these became more pronounced thereafter, consistent with a softer, less rigid framework that is more prone to macroscopic deformation (Figure 7G,H). The contrast between MatC and D at this scale already suggests that lignin contributes to mechanical rigidity and resistance to large-scale distortion.

#### 3.3.3. SEM Analysis: Fibres and Particles Before and After Soaking

**Figure 8:**
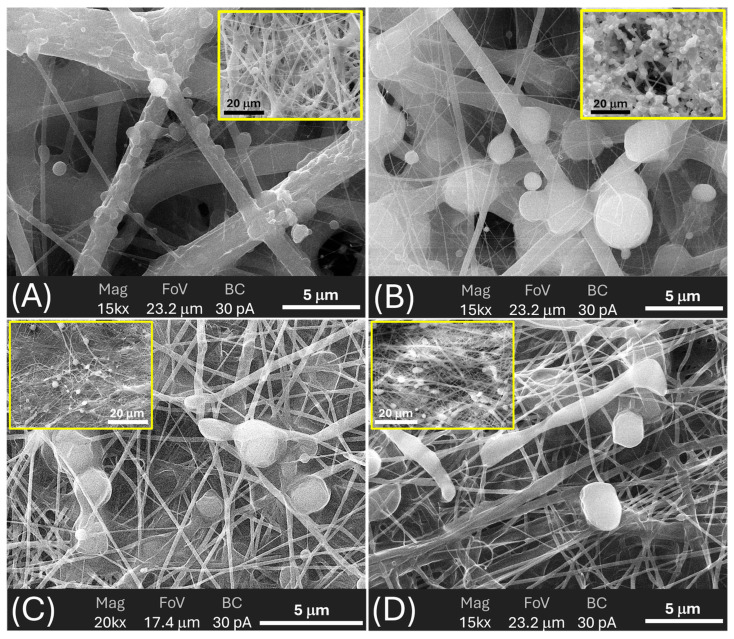
Comparison of SEM images of pristine nanostructured scaffolds (**A**) MatA, (**B**) MatB, (**C**) MatC, and (**D**) MatD. High-magnification images are shown at 15k× for MatA, MatB, and MatD, and at 20k× for MatC; the insets display the corresponding structures at lower magnification (5k×).

**Figure 9:**
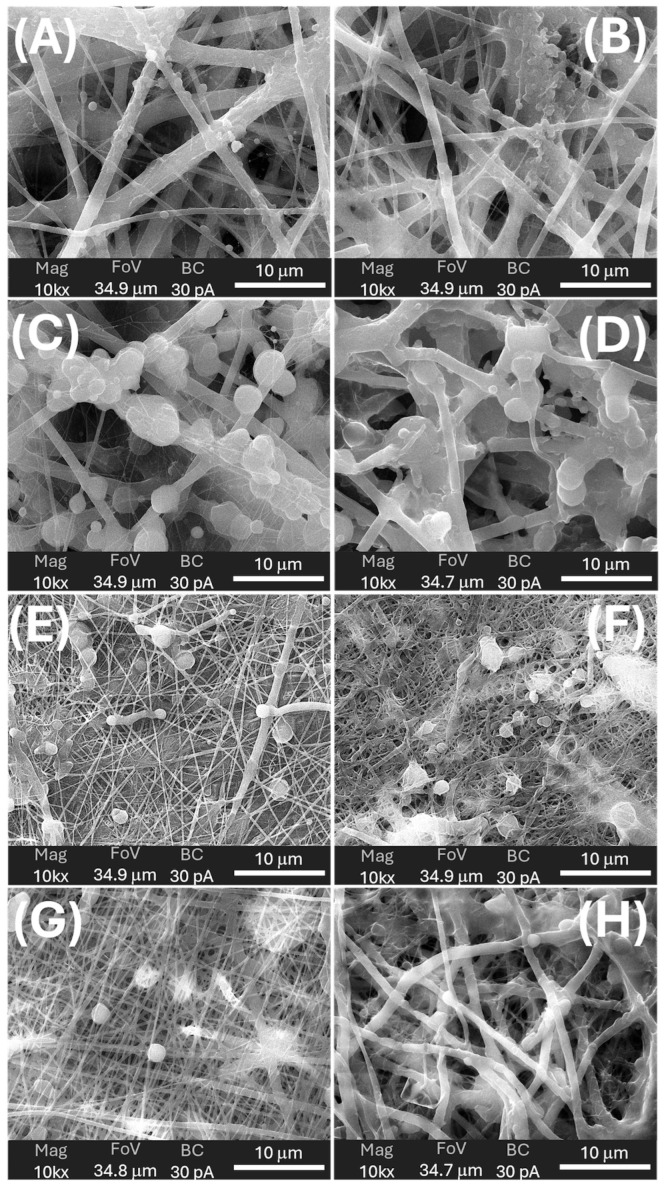
Comparison of nanostructured scaffolds MatA, MatB, MatC, and MatD before (**A**,**C**,**E**,**G**, respectively) and after (**B**,**D**,**F**,**H**, respectively) 14-day soaking in the 0.11 M phosphate buffer (pH 7.4) at 37 °C as observed by scanning electron microscopy (10k×).

SEM provided a detailed view of the multi-scale morphology of each scaffold, allowing fibre and bead dimensions to be quantified (Table 3) and their evolution on prolonged immersion in 0.11 M phosphate buffer (pH 7.4) at T_37_ to be assessed (Table 3). Figure 8 provides a multiscale morphological characterisation of the pristine scaffolds, combining low-magnification images (to visualise the overall fibre mat architecture and fibre distribution) with high-magnification images (to highlight surface features, bead-like depots, and fibre-particle interactions). In contrast, Figure 9 focuses specifically on the structural evolution after phosphate buffer immersion. For this comparative analysis, an intermediate magnification (10,000×) was deliberately selected to simultaneously evaluate fibre integrity, surface erosion, and changes in particle number and morphology under consistent imaging conditions.

- ***MatA (PHB+HS-KL fibres, PHB+GP-PP beads).*** The pristine MatA consisted of a randomly oriented PHB fibre network (mean diameter ≈ 1.0 µm, CV ≈ 2%) decorated by relatively small PHB-based particles (mean area ≈ 1.0 µm^2^, CV ≈ 42%) distributed along fibres of different sizes (Figure 8A and Figure 9A; Table 3). The overview images showed an open network with visible inter-fibre voids and bead-like elements anchored to both thicker fibres and very thin nanofibres, indicating strong adhesion between electrosprayed PHB particles and electrospun PHB fibres (Figure 8A inset). After 14 days in 0.11 M phosphate buffer (pH 7.4) at T_37_, fibres in MatA were clearly swollen: the mean diameter increased by ~32%, and inter-fibre spaces narrowed, consistent with water uptake and partial relaxation of the PHB network (Figure 9B). In contrast, the average particle area and particle roundness changed only minimally (~6%), and total particle area increased slightly (~3%), indicating that PHB beads behaved as soft, shape-preserving depots rather than densifying entities (Table 3). Morphologically, MatA thus evolves towards a more compact fibrous mesh with stable, small PHB depots.
- ***MatB (PHB+HS-KL fibres, large PCL+GP-PP beads).*** In MatB, PHB fibres (mean diameter‚ ≈1.2 µm) formed a porous, multi-diameter network similar in scale to that of MatA but interspersed with large PCL-based spherical particles (mean area ≈ 7.6 µm^2^), often partially fused or embedded in a surrounding polymer matrix (Figure 8B and Figure 9C; Table 3). At low magnification, these beads dominated the architecture, producing a pronounced fibre-bead multi-scale structure (Figure 8B inset). Very thin PCL nanofibres, originating from the electrosprayed PCL solution, were occasionally observed but were excluded from the diameter statistics (Figure 8B and Figure 9C). Upon 14-day soaking in 0.11 M phosphate buffer (pH 7.4), PHB fibres in MatB swelled moderately (+17%), and some large fibres showed surface roughening or local damage, suggesting structural relaxation and mild degradation (Figure 9D; Table 3). The small PCL nanonet visible in the pristine material was no longer detectable, suggesting its collapse or dissolution. The most striking change concerned the PCL particles: their mean area and total particle area both decreased by ~36%, while roundness remained essentially constant. This pattern indicates significant in-place densification and shrinkage of PCL depots rather than their detachment. MatB, therefore, shifts from a fibre-supported network with large, soft depots to a structure with swollen fibres and more compact PCL beads.
- ***MatC (PHB+PCL fibres with HS-KL+GP-PP, PCL+HS-KL+GP-PP beads-on-string).*** MatC was produced by co-electrospinning PHB and PCL solutions containing both HS-KL and GP-PP, yielding a single, integrated fibrous architecture in which PHB and PCL fibres were physically entangled (Figure 8C and Figure 9E). The mean fibre diameter was much smaller than in A and B (≈0.23 µm, 3-5-fold thinner), and the network appeared dense and homogeneous (CV ≈ 26%) (Table 3). Rounded and ellipsoidal features along PCL fibres were interpreted as bead-on-string structures (mean area ≈ 0.8 µm^2^, CV ≈ 58%), providing a fine population of depots embedded in the fibrous matrix. Top-view images emphasised a compact, membrane-like appearance with limited apparent surface porosity (Figure 8C inset). After 14-day soaking in 0.11 M phosphate buffer (pH 7.4), fibre diameter increased only slightly (+5%), and fibres showed limited coalescence and mild surface roughening, confirming a restrained swelling behaviour (Figure 9F). The average bead area remained essentially unchanged (+0.5%), but total bead area dropped by about 22%, indicating the loss or detachment of a fraction of bead-on-string segments rather than bead shrinkage (Table 3).

- Roundness increased modestly (+5%), suggesting that the remaining beads were slightly smoothed. Overall, MatC retains a compact fibrous backbone with a reduced but still finely distributed bead population, the main morphological change being a selective loss of PCL(HS-KL+GP-PP) depots rather than gross densification.
- ***MatD (PHB+GP-PP and PCL+GP-PP fibres, PCL+GP-PP beads, and no lignin).*** MatD, co-electrospun from PHB+GP-PP and PCL+GP-PP solutions, comprised an open, highly entangled fibrous mesh with thin fibres (mean diameter ≈ 0.28 µm) and PCL-based beads (mean area ≈ 2.8 µm^2^) that were less embedded and more superficially located than in MatC (Figure 8D and Figure 9G; Table 3). Low-magnification views revealed a looser network with greater apparent porosity and lower internal cohesion (Figure 8D inset). Prolonged soaking in 0.11 M phosphate buffer (pH 7.4) for 14 days induced the most dramatic changes among all scaffolds. Fibre diameters increased by about 175%, and the finer nanofibrous elements largely disappeared, replaced by thickened, swollen strands and partially collapsed bundles (Figure 9H; Table 3). The mean bead area and total bead area decreased by ~23%, and bead roundness dropped (~−7%), consistent with notable surface erosion and bead densification within a strongly plasticised network. Morphologically, MatD evolves into a swollen, partially collapsed structure with fewer, more irregular depots and a substantially altered pore architecture.

#### 3.3.4. Role of Architecture on Porosity and Transport Pathways

SEM observations of the four matrices suggest that assembly of fibres and depots into 3D architectures can control porosity, pore connectivity, and the transport pathways available to water and solutes. In ***MatA***, an open PHB fibrous mesh is decorated with relatively small PHB+GP-PP particles. Porosity and inter-fibre channels are abundant. Fibres carry HS-KL, whereas GP-PP is stored in separate particles, which act as soft depots embedded in a PHB gate. In ***MatB***, PHB+HS-KL fibres form a structural “skeleton” interspersed with large PCL+GP-PP beads. The bead inventory is high, and the particle-to-fibre size ratio is large, so PCL depots are expected to dominate both *mid* and *late* release once hydrated, whereas PHB+HS-KL fibres constrain overall swelling. ***MatC*** combines PHB and PCL fibres with smaller PCL beads in a more compact, intertwined network. Because both fibres and beads carry HS-KL+GP-PP, the architecture contains many parallel HS-KL-GP-PP depots distributed throughout a relatively tight mesh. ***MatD*** has a similar fibre-and-bead layout, but with GP-PP only. The absence of HS-KL allows polyphenols to plasticise both PHB and PCL to a greater extent, predisposing the network to greater swelling, pore narrowing, and bead compaction during prolonged hydration.

From a transport perspective, fibres provide long-range pathways and define pore throats, while beads and bead-on-string segments are expected to act as local reservoirs. Swelling of fibres tends to narrow pores and increase tortuosity, whereas bead shrinkage or loss can decrease depot volume or locally open channels. The balance between these opposing trends—modulated by polymer phase, HS-KL placement, and GP-PP composition—ultimately generates the different *burst–mid–late* profiles observed at T_A_ and T_37_.

#### 3.3.5. Role of Matrix Components in Scaffold Architecture and Stability

The four scaffolds described above by SEM observations (Figure 8 and Figure 9; Table 3) confirm that the three main components, i.e., carrier polymers, lignin, and polyphenols, contributed in distinct and complementary ways to the final architecture and its stability in water.

- ***Carrier polymers (PHB* vs. *PCL****)*: Comparing the four scaffolds highlights the distinct roles of PHB and PCL as carrier phases. PHB-based fibres in Mats A and B formed the thickest backbones and exhibited moderate swelling at T_37_, whereas the mixed PHB/PCL fibres in Mats C and D were initially much thinner and thus more prone to dimensional change when plasticised. However, the presence or absence of lignin and GP-PP strongly modulated this tendency: MatC, with HS-KL+GP-PP in both PHB and PCL phases, showed only minimal fibre thickening, while MatD, with GP-PP only, displayed extreme swelling. PCL-based depots were systematically larger than PHB-based ones (MatB > MatD > MatC > MatA) and, in MatB and MatD, underwent clear shrinkage on 14-day soaking in 0.11 M phosphate buffer (pH 7.4), indicating significant densification. In MatC, by contrast, bead size was preserved, and the reduction in total bead area resulted from loss rather than compaction.
- **Role of lignin:** Lignin influenced both morphology and stability. In fibres, its presence in MatA–MatC was associated with limited swelling upon 14-day soaking, whereas the lignin-free MatD exhibited pronounced fibre thickening and partial collapse of the nanofibrous network. This is consistent with lignin stiffening the amorphous phase of PHB and PCL and resisting water-driven expansion. In beads, the effect was more nuanced. PHB+GP-PP depots in MatA (no lignin) retain their size; PCL+GP-PP depots in MatB (no lignin) shrank markedly; PCL(HS-KL+GP-PP) beads in MatC largely retained their size but suffered some detachment.
- **Role of polyphenols:** Polyphenols behave as both cargo and internal plasticiser. When they are widely distributed in fibres and beads without lignin (MatD), their largely hydrophilic character favours water uptake and chain mobility, leading to strong fibre swelling, bead erosion, and a looser, partly collapsed architecture (pore narrowing and increased tortuosity). When co-localised with lignin (MatC), they instead contributed to more compact fibres and beads, consistent with the formation of lignin–polyphenol complexes reported in the literature. In depots, GP-PP combined with PCL generates very large beads in MatB that subsequently densify on 14-day soaking at T_37_, whereas GP-PP co-loaded with lignin in PCL beads (MatC) yields smaller, dimensionally stable depots (for HS-KL-GP-PP complexes), whose main change is partial loss from the web rather than shrinkage, with bead loss providing an additional pathway for cargo release. In PHB+GP-PP beads (MatA), the GP-PP load does not drive significant compaction, and the depots are small and soft.

Taken together, the morphological data indicate that: (i) ***PHB*** primarily defined the stiffness and connectivity of the fibrous framework (porosity), whereas ***PCL*** provided high-capacity depots whose size and evolution (stable, shrinking, or detachable) differed across architectures and were later reflected in the *mid* and *late* components of GP-PP release; (ii) ***Lignin*** acted as a structural stabiliser in fibres and as a modifier of depot behaviour: it limited fibre swelling and promoted depot integrity, but could shift the balance between shrinkage and loss; (iii) ***Polyphenols*** exhibited a location-dependent effect: when present alone (GP-PP-only architectures), they plasticised and destabilised the network; when complexed with lignin, they contributed to the formation of more rigid and metered depots.

Hence, each matrix approached the release tests with different features, which precisely translated into the observed *burst*, *mid*, and *late* polyphenol-release behaviours (Section 3.4 and Section 3.5): MatA as a PHB scaffold with stable small depots; MatB as a PHB backbone filled with large, densifying PCL depots; MatC as a compact PHB/PCL web with small, lignin-rich depots, some of which could detach; and MatD as a highly swellable GP-PP-rich network.

### 3.4. Interactions Between Nanohybrid Scaffolds and Water

Here, we link early wetting (water contact angle and water uptake) at T_A_ with the longer-term structural evolution of the architectures, including the stress test at 37 °C. Water uptake is the first step that activates polyphenol release from the scaffolds.

#### 3.4.1. Early Wetting and Hydration (0–10 min, T_A_)

**Figure 10:**
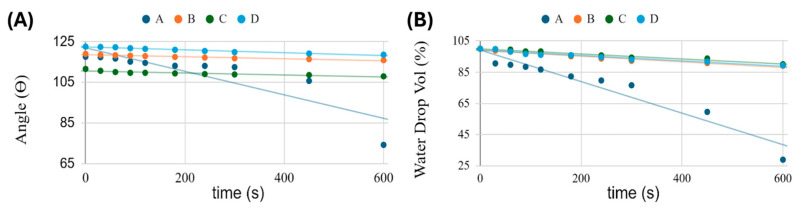
Time-dependence of water contact angle (**A**) and water droplet-volume variation (**B**) for the various scaffolds (A_blue_ = MatA, B_orange_ = MatB, C_green_ = MatC, and D_light blue_ = MatD), illustrating their different wetting and water absorption behaviours over a 600 s (10 min) period.

Dynamic water contact angle (WCA) and droplet-volume (WDV) decrease measurements at T_A_ were used to explore the outer skin of each scaffold as it hydrated in the first minutes. It is well known that both surface chemistry and roughness control wetting behaviour and can strongly influence a material’s WCA [103,104,105,106]. At deposition (t_0_), all matrices showed high WCA values (≈112–123°) (Figure 10A), consistent with rough, porous, and overall hydrophobic electrospun ± electrosprayed surfaces (SEM micrographs in Figure 8 and Figure 9). Such elevated contact angles, combined with the fibrous morphology observed by SEM, are indicative of a Cassie–Baxter-like wetting regime typically associated with partial air entrapment beneath the droplet.

Over the following 10 min, however, their trajectories diverged (Figure 10A,B). For MatB, MatC, and MatD, the WCA decreased only slightly (≈3°), while the WDV decreased by about 10%. The similar and limited changes observed for both parameters suggest that the droplet mainly remained on the surface, with the volume reduction largely attributable to evaporation rather than to significant liquid penetration into the scaffold. Such behaviour is consistent with a droplet with a largely pinned contact line and only modest superficial hydration [107,108]. In contrast, MatA showed a marked decrease in WCA (~118° to ~75°) and a ~70% apparent droplet-volume loss. The simultaneous and pronounced decrease in both WCA and WDV clearly indicates a transition from a predominantly non-wetting regime toward progressive liquid infiltration into the porous network. Evaporation alone cannot account for such a large volume change [104,106,107,109], indicating significant capillary uptake into the scaffold beneath the droplet and a partial Cassie-to-impregnating/Wenzel transition [103,105,106].

This behaviour is consistent with the matrix architectures and additive localisation described in Section 3.2 and Section 3.3. MatA consists of an open PHB+HS-KL fibrous network decorated with small PHB+GP-PP beads (Figure 8A and Figure 9A). Both lignin-containing fibres and GP-PP-loaded particles are accessible at or near the surface. HS-KL introduces polar sites that facilitate hydration, while the small PHB+GP-PP depots are not locked into HS-KL–GP-PP complexes and remain relatively soft. Under a droplet, water can rapidly penetrate into these near-surface domains, swell the fibres locally, and fill air pockets, thereby increasing the solid–liquid contact area and significantly lowering the apparent WCA.

In MatB, MatC, and MatD (Figure 8B–D and Figure 9C,E,G), the surface “seen” by the droplet is more sluggish on this timescale. In MatB, large PCL+GP-PP beads dominate the interface and hydrate more slowly; HS-KL is confined to PHB fibres that are only partially exposed. In MatC, HS-KL and GP-PP are co-located in both PHB and PCL phases, forming complexes that stiffen the amorphous regions and suppress rapid swelling. In MatD, GP-PP is present throughout PHB and PCL fibres and beads; however, during the first 10 min at ambient conditions, the hydrophobic backbone and limited chain mobility still prevent significant volumetric rearrangements. As a result, WCA and WDV for MatB-MatD remain essentially in an evaporation-dominated regime over 10 min.

In summary, early WCA and droplet-volume data indicate that MatA is intrinsically prone to rapid near-surface hydration and restructuring, whereas MatB-MatD remain comparatively inert on the minute timescale. As shown later (Section 3.5 and Section 3.6), this short-term “reactivity” is mechanistically informative but not directly predictive of the long-term release hierarchy.

#### 3.4.2. Long-Term Hydration at Ambient Temperature: Conceptual Extrapolation

**Figure 11:**
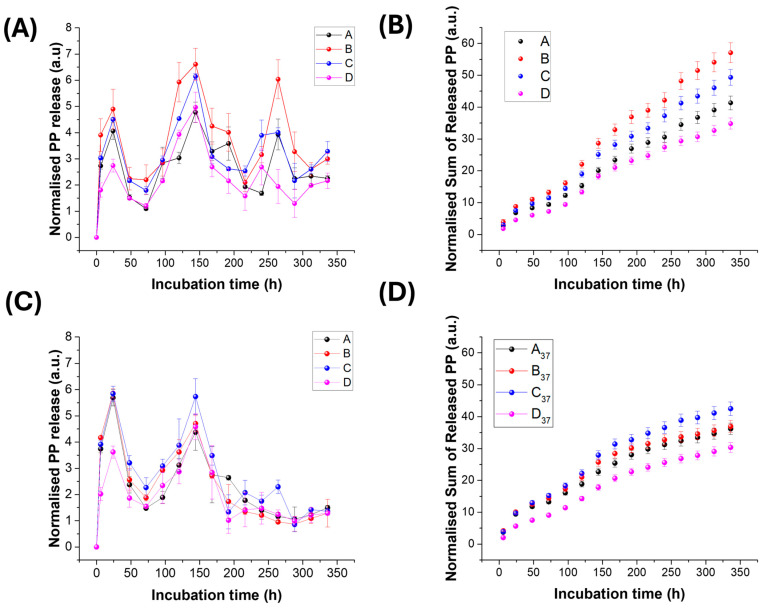
Normalised polyphenol release as a function of time for each scaffold (A_black_ = MatA, B_red_ = MatB, C_blue_ = MatC, and D_magenta_ = MatD) at ambient temperature (**A**) and 37 °C (**C**), and normalised cumulative release of polyphenols over time from the various matrices at ambient temperature (**B**) and 37 °C (**D**).

Direct morphological measurements were performed after 14 days of immersion at T_37_, whereas at T_A_, only the first 10 min were monitored using WCA and droplet-volume analysis. Nevertheless, combining early wetting at T_A_ with the T_37_ morphology data (Table 3; Figure 9) and the T_A_ vs. T_37_ release profiles provides a qualitative picture of how the architectures likely evolve under prolonged hydration at T_A_. Thus, the following interpretation is grounded on (i) dynamic WCA and droplet-volume analysis at T_A_ (Figure 10A,B), (ii) UV–Vis quantified release profiles at T_A_ (Figure 11A,B), and (iii) the T_37_ morphological reference dataset (Figure 9; Table 3).

Polymer physics suggests that the same processes observed at T_37_—fibre swelling, secondary crystallisation (MAF-to-RAF conversion), depot densification or detachment—also occur at T_A_, but more slowly and to a smaller extent, because chain mobility and diffusivity are lower. This interpretation is consistent with the systematic finding that, across all matrices, *total* polyphenol release and *late tail* areas are larger at T_A_ than at T_37_: after 14 days at T_A_, the networks remain less tightly packed and retain more accessible amorphous pathways for diffusion.

Starting from the early wetting hierarchy (MatA ≫ MatB ≈ MatC ≈ MatD), one can infer the following qualitative evolution at T_A_:

- ***MatA***—The PHB+HS-KL fibrous backbone with small PHB+GP-PP depots is expected to enter a moderately swollen state, with fibres thickening and inter-fibre pores narrowing, but without strong depot densification. The overall morphology should remain relatively open compared with its T_37_ counterpart, supporting a balanced *burst*–*mid*–*late* profile with a modest *total* release.
- ***MatB***—Large PCL+GP-PP depots that sharply densify at T_37_ will hydrate and reorganise more slowly at T_A_, so secondary crystallisation and volume loss are likely incomplete after 14 days. PHB+HS-KL fibres will still swell to some extent, but pore constriction will be less pronounced than at T_37_. This is compatible with MatB showing the highest *total* and *late* release at T_A_: the depots are plasticised but not yet fully compacted, and a substantial mobile amorphous volume remains.
- ***MatC***—At T_37_, MatC shows minimal fibre swelling and a reduction in total bead area mainly due to detachment of some PCL(HS-KL+GP-PP) bead-on-string segments. At T_A_, HS-KL-GP-PP complexes will still limit swelling and chain mobility, while mechanical stresses are milder; fibre diameters should increase only slightly, and bead detachment should be less extensive. The hydrated architecture after 14 days at T_A_ is therefore expected to stay close to the original, compact state, with a well-preserved population of HS-KL-GP-PP depots. This matches the high *total* release and particularly strong late contribution observed for MatC at T_A_.
- ***MatD***—The GP-PP-only PHB/PCL network that swells dramatically and partially collapses at T_37_ will also move towards a swollen, more tortuous morphology at T_A_, but with less extreme pore closure. Fibres will still thicken more than in the lignin-containing matrices, and PCL+GP-PP beads will densify, yet the lower temperature implies reduced swelling and compaction. This is consistent with MatD remaining the weakest releaser at both temperatures: even under gentler conditions, a sizeable fraction of GP-PP remains trapped in a highly plasticised, partially collapsed network.

Conceptually, then, prolonged hydration at T_A_ preserves the relative structural ranking seen at T_37_ -MatC compact and robust, MatD swollen and fragile, MatA and MatB in between—but with smaller deformations and more open diffusion pathways, explaining the larger *total* and *late* releases at T_A_ for all matrices.

#### 3.4.3. Morphological Evolution Under Prolonged Hydration (14 d, 37 °C)

The 37 °C immersion data provide a quantitative reference for how the scaffolds age under more demanding thermal conditions. After 14 days in buffer at T_37_, all matrices showed signs of structural reorganisation, but to varying degrees (Figure 9; Table 3).

Fibre diameters followed a clear swelling hierarchy: MatC exhibited the smallest increase (~5%), MatB and MatA showed moderate thickening (~17% and ~32%, respectively), and MatD underwent extreme swelling (~175%). This pattern is consistent with the roles of lignin and polyphenols described in Section 3.3. Lignin-containing fibres (MatA–MatC) are partially stabilised by π–π and H-bond networks with PHB/PCL, which restrain expansion, while GP-PP-only fibres in MatD are strongly plasticised and draw in much more water. Within the lignin-containing group, the PHB/PCL blend of MatC appears least prone to water-driven expansion (due to extensive HS-KL–GP-PP complexation), whereas the PHB-only network of MatA is more responsive.

Bead populations also evolved in matrix-specific ways. In MatA, PHB+GP-PP depots retained their mean size and total area, indicating that they remained soft but did not densify appreciably (full quantitative data are reported in Table 3). In MatB, large PCL+GP-PP beads shrank markedly in both mean and total area, consistent with in situ densification driven by GP-PP loss, secondary crystallisation, and collapse of residual microvoids. In MatC, PCL(HS-KL+GP-PP) beads showed almost unchanged mean area but a substantial reduction in total bead area (~22%), implying that a fraction detached from the fibrous web rather than shrinking in place. In MatD, PCL+GP-PP beads shrank in both mean and total area (~23%), but now within a highly swollen fibre web, indicating depot compaction inside a gel-like, GP-PP-rich matrix (Table 3).

These coupled changes in fibre swelling and depot evolution have direct consequences for transport pathways and, ultimately, for release behaviour (Section 3.5). Moderate swelling with stable depots (MatA) yields a conservative, self-limiting profile; strong depot densification (MatB) front-loads release and weakens the late tail; minimal swelling with partial depot loss (MatC) preserves a compact network yet shifts some release to earlier times while maintaining the best late fraction; and extreme fibre swelling plus depot compaction (MatD) produces a tortuous, partially collapsed architecture that severely restricts overall polyphenol discharge. In the following section, these hydration and ageing patterns are explicitly linked to the *burst*, *mid*, and *late* release components at both T_A_ and T_37_.

### 3.5. Polyphenol Release from the Biohybrid Nanostructures

The electrospun ± electrosprayed scaffolds were designed as biohybrid matrices that release a grape-pomace polyphenol (GP-PP) extract in a controlled manner. Four architectures were tested (MatA-D), differing in polymer phase (PHB vs. PCL blends), lignin (HS-KL) content and localisation, and GP-PP distribution between fibres and bead-like depots (Table 1). Polyphenol release was quantified at T_A_ and T_37_, and the resulting profiles were analysed in terms of *burst*, *mid*, and *late* phases, *total* released area, and t_50_, as described in Section 2.7. A schematic of typical CRF release profiles under unimodal/monomodal, bimodal, and multimodal models is shown in Figure S4, which also displays the different release phases [110]. Typically, the cumulative curves of CRF vs. SRF vs. traditional fertilisers exhibit sigmoidal shapes [111] (Figure 5). Similarly, the cumulative curves of the matrices created here were sigmoidal, with visible inflexion points, confirming that the scaffolds behave as CRF-like systems rather than as simple diffusive slabs.

#### 3.5.1. Release Profiles at Ambient Temperature (T_A_)

At T_A_, all matrices showed a clear multimodal release with a *burst* phase, containing a modest peak at ≈24 h, a *mid* phase, containing the second pronounced peak centred at ~120–144 h, and the *late tail*, containing the third peak at ~240–264 h plus the terminal shoulder at ~336 h (Figure 11A). In terms of *total* GP-PP released (integrated area), the ranking at T_A_ was as follows: MatB > MatC > MatA > MatD (≈57.1 > 49.4 > 41.4 > 34.8 a.u., respectively) (Table 4). The early *burst*, *mid*, and *late* regions followed the same order in absolute area (MatB > MatC > MatA > MatD), whereas t_50_ values clustered between ≈140 and 148 h, with MatA being the slowest and MatD the fastest to reach 50% of its own cumulative release, hence highlighting that MatB maximises throughput, MatC balances throughput and control, MatA constrains delivery, and MatD illustrates that GP-PP-only networks are poorly controlled-release carriers. The cumulative curves of the matrices exhibited a sigmoidal shape with visible inflexion points, corresponding to the release peaks described before (Figure 11B).

**Figure 12:**
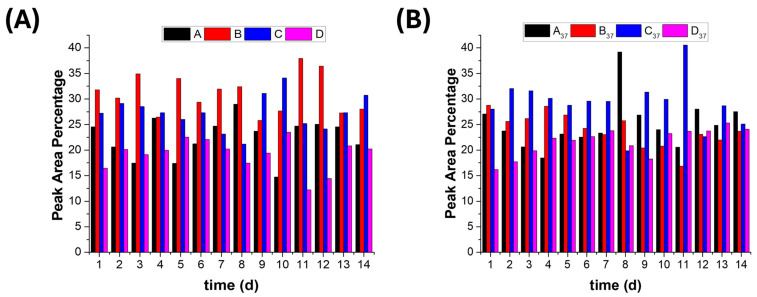
The day-by-day contribution of the various matrices (A_black_ = MatA, B_red_ = MatB, C_blue_ = MatC, and D_magenta_ = MatD) along the 14-day incubation period, calculated as the percentage of the UV-VIS absorbance of the incubation solutions of each matrix per day relative to the sum of the UV-VIS absorbance of all the fabrics on the same day, at both T_A_ (**A**) and T_37_ (**B**).

On a day-by-day basis, the normalised daily release trends at T_A_ (Figure 12A) show that MatB was the dominant contributor on roughly half of the sampling days (7/14), MatC led on the first 3 days, while MatB and MatC contributed comparably, on average, and more than other matrices, on the remaining days. MatD is consistently the weakest contributor (with the lowest daily share on 12/14 days), whereas MatA occupies an intermediate position throughout the test period. This confirms that the higher integrated areas of MatB and MatC reflect genuinely stronger performance across the entire two-week window rather than being driven by a single transient event.

- ***MatA*** (PHB+HS-KL fibres + PHB+GP-PP depots with no HS-KL-GP-PP co-location). MatA delivered an intermediate *total* amount (41.4 a.u.; Table 4), with a modest *burst*, a moderate *mid* contribution, and a medium *late tail*. The cumulative curve is clearly multimodal but with a relatively shallow slope (Figure 11B). The latest t_50_ (≈148 h; Table 4) of MatA reflects diffusion through a PHB-only network in which HS-KL stabilises the fibres but does not directly meter the GP-PP depots. Release is governed by slow diffusion from PHB beads and by the porosity set by fibre diameter and bead coverage. Overall, MatA behaves as a conservative, low-dose scaffold with a relatively delayed response.
- ***MatB*** (PHB+HS-KL fibres + large PCL+GP-PP depots with segregated HS-KL and GP-PP). At T_A_, MatB was the most productive matrix overall (*total* = 57.1 a.u.; Table 4), combining the largest *burst* (13.3 a.u.), *mid* (23.7 a.u.), and *late* (20.2 a.u.) areas. Although its *burst* was the strongest among the four matrices, it still represented only a minority of the *total* release, with most of the mass delivered in the *mid* and *late* windows. The cumulative curve for B therefore rises steeply but not explosively and clearly dominates those of the other scaffolds (Figure 11B). This behaviour reflects its architecture: PHB+HS-KL fibres provide a relatively stable supporting network, while the large PCL+GP-PP depots act as high-capacity, MAF-rich sources that are easily hydrated and drained at T_A_. Because HS-KL resides only in the PHB phase, it primarily stabilises the fibrous mesh rather than metering GP-PP within the PCL beads; as a result, MatB functions as a high-throughput system with strong *mid* and *late* contributions, suitable where a relatively high dose of polyphenols over 1–2 weeks is desired, even if fine control over tail “quality” is less critical.
- ***MatC*** (PHB+PCL fibres and PCL beads, all with HS-KL+GP-PP co-located). MatC released somewhat less GP-PP than B (49.4 a.u.; Table 4) but with a smoother profile and a particularly strong late contribution. *Burst* and *mid* phases were high, and the *late tail* was second only to MatB in absolute terms but largest in relative terms (*late*/*total*). The cumulative curve of MatC tracks just below that of MatB (Figure 11B). Here, HS-KL and GP-PP share the same microdomains in both PHB and PCL phases, so HS-KL-GP-PP complexes mediate desorption and stabilise labile species (anthocyanins and flavonols). The architecture behaves as a system of parallel depots distributed in fibres and beads, providing a broad, well-structured *burst*–*mid*–*late* sequence. At T_A_, MatC is thus slightly less productive than MatB in total mass but more controlled and better suited when the quality and persistence of the *late* phase are important.
- ***MatD*** (PHB+PCL fibres and PCL beads, with GP-PP only and no HS-KL). MatD, despite the highest nominal GP-PP loading, gave the lowest *total* release at T_A_ (34.8 a.u.; Table 4), with the smallest *burst* and *late* areas. The cumulative curve lies clearly below those of the other systems (Figure 11B). The absence of HS-KL means that GP-PP primarily acts as a plasticiser for PHB and PCL; the network swells and softens rather than forming well-defined, stabilised depots. As a result, the scaffold is structurally quite responsive but functionally inefficient: pathways become tortuous, and a significant fraction of GP-PP remains trapped. MatD is therefore the least effective matrix at T_A_, despite its apparent “capacity”.

Overall, the T_A_ data show that long-term performance depends far more on polymer phase, depot morphology, and HS-KL placement than on loading alone. MatB maximises throughput; MatC balances throughput and control; MatA constrains delivery; and MatD demonstrates that GP-PP-only networks are poorly controlled-release carriers.

A summarised description of matrix-by-matrix features at T_A_ is reported in Table S5.

#### 3.5.2. Release Profiles at 37 °C

- Incubation at 37 °C represents a realistic warm-soil scenario for shallow Mediterranean horizons and a useful stress test for the scaffolds (§S6) [72,73,74,75,76,77,78,79,80,112,113]. At this temperature, water and solute mobility increase, but polymer reorganisation (swelling, secondary crystallisation, and depot densification or detachment) is also accelerated. Across all matrices, peak fitting and cumulative curves revealed three general trends (Figure 11; Table 4): (i) *total* release decreased relative to T_A_ (≈−12 to −35%, depending on the matrix); (ii) *late tails* were strongly suppressed (≈−34 to −66%), so that the clear multimodal profiles were compressed; and (iii) t_50_ shifted to earlier times (≈103–125 h vs. 140–148 h at T_A_), indicating that the PP released tends to do it sooner, before structural tightening fully develops. The ranking at T_37_ was: (i) *total *area: *MatC > MatB > MatA > MatD*; (ii) *burst *and *mid* areas: *MatC > MatB > MatA > MatD*; (iii) *late *area: *MatC > MatA > MatD > MatB*; and (iv) t_50_: *MatB < MatC < MatA < MatD*. The GP-PP release profile (Figure 11C) exhibited a multimodal shapeas well in this case, with a *burst* phase, containing an increased peak at ≈24 h, relative to T_A_, the *mid* phase, containing the second peak at ~120–144 h, and the *late tail*, containing the third peak at ~240–264 h plus the terminal shoulder at ~336 h. The corresponding cumulative release trends at 37 °C (Figure 11D) further illustrate the matrix-dependent differences in total release and late-phase persistence under warmer conditions.
- On a day-by-day basis, the normalised daily release trends at T_37_ (Figure 12B) show that MatC was the dominant contributor from day 2 to day 7 and from day 9 to day 13, while MatA led on days 8, 12, and 14. MatA, MatB, and MatC exhibited a comparable burst (day 1). MatD, unlike what was observed at T_A_, presented a normalised daily release comparable to that of MatA and MatB from day 3 onward (except on days 8 and 9). This confirms that, in this case, the higher-integrated areas of MatC reflect stronger performance over the two-week period rather than being driven by a single transient event.
- ***MatA***—At T_37_, MatA exhibited a modest reduction in *total* release (~−12%) and a marked decrease in *late*-area (~−43.5%), while its *burst* increased by 25.3% relative to T_A_, and t_50_ decreased from 148.3 h to 118.5 h (Table 4). PHB+HS-KL fibres swell and undergo secondary crystallisation, narrowing diffusion pathways, while PHB+GP-PP depots remain relatively soft but progressively less accessible. The profile becomes more front-loaded (larger early contribution, weaker tail), consistent with a scaffold that reacts quickly to warming but then self-limits further release.
- ***MatB***—MatB was the most temperature-sensitive system. *Total* release dropped by about one-third, and the *late tail* decreased by roughly two-thirds compared with T_A_ (Table 4). The *burst* and mainly *mid* phases were also weakened. t_50_ shifted from 143.6 to 103.2 h. Morphologically, PHB+HS-KL fibres swell, whereas large PCL+GP-PP beads densify and shrink, thereby reducing the accessible MAF volume. These changes push more releases earlier and sharply erode *late*-phase capacity. At T_37_, MatB therefore loses much of the advantage it had at T_A_ and no longer dominates in either *total* or *late* delivery.
- ***MatC***—MatC showed only a moderate reduction in *total* (≈−14%) and *late* (~−47%) areas, whereas both *burst* and *mid* peaks increased relative to T_A_ (Table 4). t_50_ decreased but remained intermediate. Structurally, fibres swelled only slightly, preserving pore connectivity, and PCL(HS-KL+GP-PP) beads tended more to detach than to shrink in place. HS-KL-GP-PP co-location in both PHB and PCL phases continued to meter desorption and stabilise flavonols, stilbenes, and anthocyanins. As a result, even under stress conditions, MatC maintained the highest *total* release and the strongest *late* tail in absolute terms. It thus emerges as the most robust matrix at 37 °C.
- ***MatD***—For MatD, *total* release decreased by only ~13%, but it delivered the lowest overall GP-PP, with the smallest relative drop in the *mid* area and moderate *late* suppression (Table 4). However, MatD still delivered the lowest overall GP-PP at T_37_. The extreme swelling of GP-PP-only fibres (~+174%) and densification of PCL+GP-PP beads (~−23%) (Table 3) produced a highly swollen, partially collapsed network with reduced porosity and high tortuosity. Release became largely access-limited and poorly controllable; the profile remained weak across all phases.
- In summary, warming to 37 °C compresses and attenuates the multimodal profiles for all matrices, but in matrix-specific ways. MatC retains both the highest *total* release and the best *late* component; MatB suffers the greatest loss in performance; MatA becomes a more conservative system; and MatD remains the least efficient.
- A summarised description of the main matrix-by-matrix features at T_37_ is reported in Table S6.

#### 3.5.3. Polyphenol Release Rates at Ambient and 37 °C Temperatures

**Figure 13:**
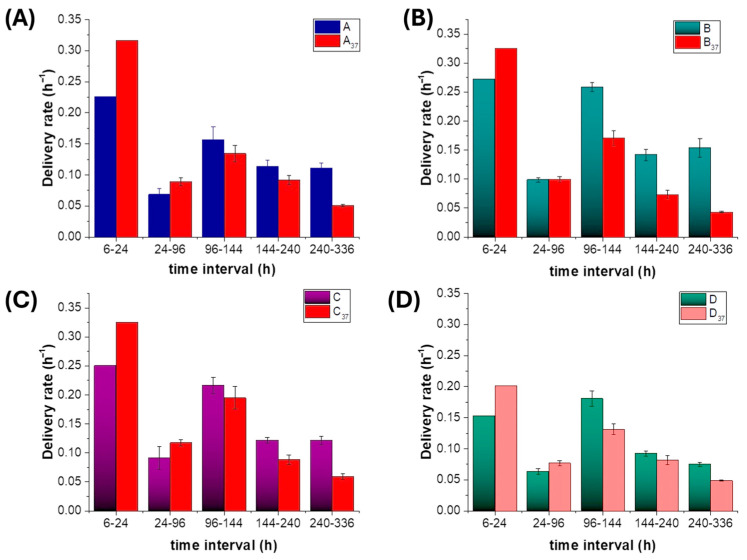
Normalised polyphenol release rates from each scaffold (**A** = MatA, **B** = MatB, **C** = MatC, and **D** = MatD), calculated at different incubation period intervals at ambient temperature and at 37 °C (e.g., A vs. A_37_, respectively).

In both datasets (T_A_ and T_37_), we also examined instantaneous release rates (Figure 13), obtained as the slope of the cumulative curves over time intervals defined by the inflexion points of the multimodal profiles (Figure 11). At T_A_, MatB and MatC exhibited the highest values. At the same temperature, all four matrices displayed higher peak release rates than at T_37_ in the *mid* interval, with MatB and MatC clearly dominating. In the *late* phase, MatA, MatB, and MatC remained constant. Differently, MatD remained rate-limited at every stage. At T_37_, all four scaffolds exhibited a higher *burst* than at T_A_. In addition, warming primarily compressed and attenuated the *mid* and *late* phases, particularly in MatB and MatD, whereas MatC remained the most productive and structurally robust system.

#### 3.5.4. Key Parameters Controlling Polyphenol Release

The differences between matrices and temperatures can be rationalised in terms of a limited set of interdependent design variables.

1. ***Polymer phase and MAF/RAF microstructure:*** Both PHB and PCL are semicrystalline polyesters with crystalline lamellae embedded in an amorphous phase. Electrospinning produces oriented fibres in which the amorphous material can be separated conceptually into a mobile amorphous fraction (MAF), which hosts most of the diffusion pathways, and a rigid amorphous fraction (RAF) at crystal interfaces, which contributes more to mechanical stiffness than to transport. PHB typically crystallises more strongly and generates a larger RAF, making it a slower, more gate-like carrier [91,92,98]. PCL, with a lower melting point and more mobile chains at both T_A_ and T_37_, offers more continuous MAF pathways and behaves as a faster depot [114,115]. In the present architectures, PHB-rich regions therefore tend to set the overall permeability and *late*-phase quality, whereas PCL-rich depots largely control throughput in the *burst* and *mid* windows.
2. ***Bead evolution: shrinkage* vs. *detachment:*** At T_37_, PCL-based beads evolved in two main ways: shrink-in-place densification (MatB and MatD) and adhesion-limited detachment (MatC). Densification reduces depot volume and mobile amorphous pathways, thereby eroding *mid*/*late* capacity, even if it shortens diffusion paths locally. Detachment instead removes some depots from the fibrous network, generating an early-release pulse while preserving the internal structure of the remaining HS-KL–GP-PP depots, thereby sustaining optimal *late*-phase release. PHB+GP-PP beads in MatA, by contrast, remained largely unchanged in size, acting as soft but increasingly shielded sources as the surrounding PHB matrix tightened.
3. ***Fibre swelling and pore architecture:*** Fibres are not merely passive supports: they act as depots when loaded with GP-PP and, more importantly, as walls that define pore size, connectivity, and tortuosity. Swelling increases fibre diameter and reduces effective pore throats [116,117], so a limited expansion can improve water access and wet the depots, whereas excessive swelling closes channels and throttles diffusion. In our system, co-electrospun PHB/PCL fibres in MatC swell least, PHB+HS-KL fibres in MatA and MatB show intermediate swelling, and GP-PP-only PHB/PCL fibres in MatD swell most, in line with their different GP-PP and HS-KL contents (Table 3), and the hydrophilic and H-bonding plasticising features of GP-PP. Thus, MatC preserves a relatively open, stable pore network that supports sustained *mid*/*late* release even at T_37_; MatA and MatB progressively tighten their pores but retain usable pathways; and MatD evolves towards a highly swollen, tortuous mesh in which transport becomes access-limited, and a large fraction of GP-PP remains trapped. Fibre swelling, therefore, acts as a second-level control on release, operating in parallel with bead densification/detachment and the chemical effects of HS-KL–GP-PP interactions.
4. ***Lignin as a physical and chemical stabiliser:*** HS-KL plays a dual stabilising role. Physically, it forms π–π and H-bond networks with PHB and PCL, which limit fibre swelling and promote secondary crystallisation in a controlled manner, thereby preserving the pore architecture—especially in MatC, where swelling is minimal even at T_37_. Chemically, when HS-KL and GP-PP are co-located (MatC), lignin provides binding sites and hydration shells that stabilise polyphenols and slow their desorption, yielding better-structured *mid/late* release and a more chemically rich *late tail*. When HS-KL and GP-PP are segregated (MatB), or HS-KL is absent (MatD), depots are more prone to uncontrolled densification, and sensitive GP-PP classes are less protected.
5. ***Co-location* vs. *segregation of HS-KL and GP-PP; GP-PP-only networks:*** The comparison between the matrices illustrates three regimes: (i) Co-location (MatC)—HS-KL and GP-PP in the same PHB and PCL domains create genuine HS-KL-GP-PP depots: swelling is restricted, depots meter release, and labile species are better preserved. This configuration supports a broad *burst*–*mid*–*late* sequence that is relatively stable to temperature. (ii) Segregation (MatA, MatB)—HS-KL in fibres and GP-PP in separate depots provide structural reinforcement and some gating, but do not directly co-stabilise the cargo. MatB exploits large PCL+GP-PP depots to maximise throughput at T_A_, but loses much of this advantage at T_37_; MatA remains more conservative at both temperatures. (iii) GP-PP-only networks (MatD)—Fibres and depots without HS-KL are strongly plasticised by GP-PP and water. Swelling and densification dominate, porosity collapses, and a substantial fraction of GP-PP remains trapped. This configuration performs poorly as a controlled-release system, even under high loading conditions.

Taken together, these observations point to a set of practical design rules for CRP-type materials based on PHB/PCL, lignin and polyphenols: combine a slower, more crystalline phase (PHB) with faster PCL depots; ensure at least one architecture in which HS-KL and GP-PP are co-located to create stabilised depots; limit GP-PP-only carrier phases; and tune bead size and adhesion so that depots contribute both to *mid* and *late* release without excessive densification or irreversible loss.

### 3.6. Integrated Mechanistic Interpretation

This section integrates the structural, wetting, and release data to identify the main design rules governing polyphenol delivery from the four biohybrid matrices.

#### 3.6.1. Early Wetting (10 min, T_A_) Versus Long-Term Release (14 Days, T_A_)

Bulleted dynamic WCA/WDV tests at T_A_ only probe during the first few minutes of the scaffold–water interaction under a sessile droplet. Under these conditions, MatA clearly stands out: its apparent WCA drops from ≈110–120° to ~75° and the WDV decreases by ~70%, whereas MatB–D show only a few degrees of WCA decrease and ~10% volume loss, values compatible with evaporation plus modest superficial hydration. Thus, at T_A_, the early wetting hierarchy is as follows: MatA ≫ MatB ≈ MatC ≈ MatD.

This picture changes when the release is viewed over a 14-day period at T_A_. The *total* GP-PP released follows the order MatB > MatC > MatA > MatD, and the *burst*, *mid*, and *late* areas all follow the same ranking in absolute terms. t_50_ is longest for MatA and shortest for MatD (MatA > MatB > MatC > MatD). Hence, the scaffold that hydrates fastest at the surface (MatA) is not the most productive releaser, whereas a matrix with little early WCA change (MatB) dominates the long-term delivery at T_A_.

Mechanistically, this decoupling reflects the difference between local surface restructuring and bulk reorganisation of the 3D network: (i) ***MatA*** combines PHB+HS-KL fibres with PHB+GP-PP beads. HS-KL and GP-PP are both present in the near-surface region, so the Cassie state collapses locally within minutes, resulting in pronounced changes in WCA/WDV. However, PHB depots do not densify, GP-PP loading is moderate, and the PHB-only framework remains relatively restrictive; the result is a modest *burst* and intermediate *mid/late* release, despite the very reactive “skin”. (ii) ***MatB*** couples PHB+HS-KL fibres with large PCL+GP-PP beads. On the 10 min timescale, water mainly contacts hydrophobic PCL surfaces and a limited area of PHB+HS-KL fibres, so WCA and WDV change only slightly. Over hours to days, however, water gradually plasticises both the PHB and PCL phases, without yet triggering strong bead densification at T_A_. Large PCL depots then deliver high *burst*, *mid*, and *late* fluxes, making MatB the most productive system in all three phases at T_A_. (iii) ***MatC*** is structurally tight at T_A_ due to the co-location of HS-KL-GP-PP in both PHB and PCL fibres and PCL beads. Osmotic water uptake and swelling are reduced, so early wetting is conservative. Yet, once hydrated, multiple HS-KL-GP-PP depots in both phases provide well-metered pathways, yielding the second-highest *total* release and the largest fractional *late* contribution. (iv) ***MatD*** contains GP-PP-only PHB and PCL fibres, as well as PCL beads. Even at T_A_, this configuration tends to swell and partially densify depots, trapping a relevant portion of the payload and limiting effective diffusion. As a result, MatD is the weakest releaser despite the highest nominal GP-PP loading.

In summary, 10 min WCA/WDV at T_A_ reports how the outer skin hydrates and restructures, whereas 14-day T_A_ release reflects how the entire fibre–bead network reorganises. A strongly “reactive” surface (MatA) does not guarantee the highest long-term release, and a seemingly inert surface (MatB) can still support the most productive *burst/mid/late* sequence once the bulk has hydrated.

#### 3.6.2. Effect of Warming to 37 °C: How Temperature Reshapes Release

At T_37_, three processes act together: (i) water and solute mobility increase; (ii) polymer reorganisation accelerates (fibre swelling, secondary crystallisation, bead shrinkage, or detachment); and (iii) thermal sensitivity of GP-PP and HS-KL-GP-PP complexes becomes relevant. Under these conditions, the hierarchy of GP-PP release becomes the following: (i) *Total* area: MatC > MatB > MatA > MatD; (ii) *Burst* and *mid*: MatC ≥ MatB > MatA > MatD; and (iii) *Late tail*: MatC > MatA > MatD > MatB; t_50_: MatB < MatC < MatA < MatD.

Thus, MatC becomes the dominant releaser at 37 °C, MatB loses much of its advantage, MatA remains intermediate, and MatD stays the weakest despite its strong swelling.

These changes can be traced back to the different morphologies and HS-KL/GP-PP placements: (i) ***MatA*** (PHB+HS-KL fibres and PHB+GP-PP beads): At T_37_, PHB+HS-KL fibres swell moderately (~32% increase in diameter), and PHB depots hydrate more rapidly. Faster diffusion enhances the *burst* area, but PHB ageing (MAF→RAF conversion, lamellar thickening) narrows pores and progressively suppresses *mid* and *late* flux. Overall, MatA becomes more front-loaded at 37 °C, with a conservative *late* tail and intermediate *total* release. (ii) ***MatB*** (PHB+HS-KL fibres, large PCL+GP-PP beads): MatB is the most temperature-sensitive system. At T_37_, PHB+HS-KL fibres swell (+17%), and PCL beads shrink and densify (≈36% in area), reducing the accessible amorphous volume and cutting off *late*-diffusion pathways. The *burst* and *mid* remain appreciable, but the *late tail* is sharply reduced, and total release drops by more than one-third compared with T_A_. MatB thus shifts from the best T_A_ performer to a matrix with fast t_50_ and a truncated tail under warm stress. (iii) ***MatC*** (PHB/PCL fibres and PCL beads with HS-KL+GP-PP co-located): Co-location of HS-KL and GP-GP-PP in both polymer phases strongly limits fibre swelling (+4.8%) and favours bead detachment (≈22% total bead area) rather than shrink-in-place. At T_37_, this architecture responds by increasing *burst* and *mid* areas (due to faster hydration and partial release from labile or detached depots) while still preserving the highest *total* release and the strongest absolute *late tail*. HS-KL-GP-PP complexes continue to meter diffusion and stabilise labile classes, so MatC emerges as the most robust, well-structured releaser under warm conditions. (iv) ***MatD*** (GP-PP-only PHB/PCL fibres and PCL beads): MatD experiences extreme fibre swelling (~+175% diameter) and bead densification (≈23% area) at T_37_, resulting in a highly swollen, partially collapsed network. Tortuosity and access limitations dominate; the *burst* remains the smallest, *mid* and *late* peaks are weak, and *total* release is the lowest at both T_A_ and T_37_. Large structural changes, therefore, do not translate into effective delivery when no lignin is present to stabilise and meter the system.

Overall, the 37 °C test shows that the apparent advantage of higher temperature for diffusion is often offset by structural tightening and depot evolution. Only the HS-KL-GP-PP co-located architecture of MatC preserves a high *total* output and a useful *late tail* under warm stress.

#### 3.6.3. Final Synthesis and Design Rules

Combining the T_A_ and T_37_ results yields a coherent set of design rules: (i) *Short-term WCA/WDV at T_A_ is mechanistic rather than predictive.* It identifies scaffolds whose surface is intrinsically prone to rapid hydration and Cassie-state collapse (MatA), but it does not, by itself, predict long-term performance. The 14-day release profiles at T_A_ and T_37_ are instead governed by bulk morphology, fibre swelling, bead evolution, and HS-KL-GP-PP localisation. (ii) *Polymer phase and morphology must be deliberately combined.* PCL beads and bead-on-string domains act as high-capacity depots and dominate throughput (especially in MatB and MatC), whereas PHB, particularly when combined with HS-KL, provides structural rigidity and gating, thereby influencing the extent of that throughput and the behaviour of *late tails*. (iii) *HS-KL-GP-PP co-location is the most effective strategy.* When HS-KL and GP-PP share the same PHB and PCL domains (MatC), HS-KL-GP-PP complexes reduce fibre swelling, stiffen the amorphous regions, stabilise labile polyphenols, and support a structured *burst–mid–late* sequence that remains robust at T_37_. When HS-KL is restricted to fibres and GP-PP to separate depots (MatB and MatA), or when HS-KL is absent (MatD), temperature-induced densification and ageing are less buffered, and *late* phases are structurally or chemically compromised. (iv) *Bead behaviour and fibre swelling act as coupled levers.* In MatB and MatD, PCL beads shrink in place at T_37_, reducing depot volume and *late* capacity; in MatC, HS-KL-bearing beads are more prone to detachment, generating an early/*mid* pulse without fully sacrificing the *late tail*. In parallel, limited fibre swelling (MatC) preserves pore architecture and transport pathways, whereas extreme swelling (MatD) closes pores and traps GP-PP in a highly hydrated but poorly permeable network.

From a practical standpoint, these rules explain why MatB is the best option for maximum short- to mid-term mass delivery at T_A_, MatC is the most reliable and chemically “clean” releaser under both T_A_ and warm conditions, MatA provides a conservative, self-braking profile, and MatD illustrates the limits of GP-PP-only carriers.

### 3.7. Potential Applications of the Biohybrid Nanostructures

The release profiles observed in this study, characterised by a modest initial burst (day 1), a pronounced mid-phase pulse (days 5–8), and a sustained late tail extending to days 10–14, define a two-week functional window rather than a season-long fertiliser curve. In Mediterranean systems, this interval overlaps transplant shock, early root proliferation, and the onset of rapid vegetative growth. During these phases, polyphenol delivery may buffer oxidative stress, stabilise cellular membranes, and modulate rhizosphere processes, potentially improving nutrient-use efficiency (NUE) rather than supplying nutrients directly. As functional metabolites rather than nutrients, polyphenols influence oxidative balance and soil-root interactions, including microbial signalling, Fe/P availability, and organo-mineral dynamics. The multimodal release pattern identified here may therefore align with temporally distinct functional demands, such as (i) early transplant stress (day 0–3), (ii) root initiation and early nutrient uptake (day 3–7), and (iii) the onset of rapid vegetative growth (day 7–14), especially under fluctuating moisture and seasonal heat stress. At a conceptual level, the four matrices exhibit release signatures that could tentatively be associated with different agronomic scenarios, depending on temperature and management conditions (Tables S6 and S7). These associations are intended as hypothesis-generating extrapolations based solely on release kinetics and matrix behaviour. Under *ambient conditions* (≈15–25 °C), ***MatB*** exhibits the highest short-term throughput and may be compatible with fast-growing leafy crops that require rapid early biomass expansion. ***MatC***, characterised by a more structured late-phase persistence and chemical stability, may be more suitable in systems where sustained antioxidant support is desirable during establishment. ***MatA*** provides a conservative, low-dose profile that could be relevant in sensitive or low-input contexts. In contrast, ***MatD*** shows limited effective release relative to loading, suggesting reduced functional efficiency in its current formulation. Under *warmer conditions* (≈32–37 °C), matrix performance reshuffles in response to thermal stability and depot evolution. ***MatC*** retains the most robust late-phase contribution and total release, indicating resilience under heat stress. ***MatA*** maintains a moderated, self-limiting behaviour. ***MatB*** becomes more temperature-sensitive due to depot densification, and ***MatD*** exhibits pronounced swelling and reduced release control. These observations reflect intrinsic matrix architecture rather than crop-specific validation. Illustrative examples for Mediterranean systems (e.g., transplanting of Solanaceae or cucurbits, short-cycle leafy crops, perennial liners) are provided as potential application scenarios, but field validation remains necessary to confirm biological efficacy and crop-specific optimisation.

## 4. Conclusions

This work tested whether eco-friendly nanotechnological products composed of electrospun ± electrosprayed PHB/PCL biohybrid scaffolds loaded with agro-waste-derived lignin and polyphenols (from hazelnut shells and grape pomace, respectively) could function as controlled-release polyphenol (CRP) systems for agricultural use. Four matrices were engineered by varying polymer phase (PHB vs. PCL), Klason lignin presence and localisation, and the distribution of polyphenols (GP-PPs) between fibres and bead-like depots. Morphology, early wetting, and long-term release at T_A_ and T_37_ were analysed using CRF-type criteria (*burst* amplitude, *burst–mid–late* structure, t_50_, temperature robustness, and *late-tail* quality).

All matrices exhibited multimodal GP-PP release at T_A_, with three peaks grouped into *burst*, *mid*, and *late* phases over ~14 days, with modest initial *bursts*, which is favourable for avoiding transient phytotoxicity and better matching plant nutrient demand. At T_A_, the PHB/PCL composite MatB (HS-KL in PHB fibres and GP-PP in large PCL beads) showed the highest *total* release and the largest *late tail*; MatC (HS-KL and GP-PP co-located in both PHB and PCL fibres and in PCL beads) ranked second in *total* but provided a smoother, better metered profile and a chemically “richer” *late* fraction (flavonols and anthocyanins); MatA (PHB+HS-KL fibres and PHB+GP-PP particles, no HS-KL–GP-PP co-location) delivered an intermediate, more conservative profile; and MatD (GP-PP-only PHB/PCL fibres and beads, no HS-KL) released the smallest amount despite the highest nominal GP-PP loading, underscoring that GP-PP loading alone does not imply effective delivery.

When the temperature was raised to 37 °C, mimicking shallow Mediterranean soils during hot periods, all profiles became more compressed (earlier t_50_) and *total* and *late* release decreased, due to PHB ageing, PCL depot reorganisation, and fibre swelling. Under these stressed conditions, MatB became the most temperature-sensitive, with strong losses in *mid* and *late* release; MatA showed a modestly increased *burst* but stronger *mid/late* gating; MatD suffered extreme swelling and depot densification with persistently weak release; in contrast, MatC remained structurally and functionally the most robust, retaining the highest *total* GP-PP release and the strongest *late tail* at T_37_, enriched in flavonols, stilbenes, and HS-KL-stabilised anthocyanins, despite PCL bead detachment.

In agronomic terms, these findings suggest that at T_A_, MatB is suitable where a high two-week GP-PP dose is desired (e.g., fast-growing or short-cycle crops in cooler or irrigated systems), whereas MatC is preferable when a slightly lower *total* amount but a better controlled, sustained, and chemically valuable *late* phase is required. Under warm conditions around T_37_, MatC clearly outperforms the other formulations in both quantity and quality of *late* release; MatA behaves as a conservative, self-limiting scaffold, and MatD remains too weak for practical use.

Beyond ranking individual formulations, the study yields general design rules for CRP materials from agro-industrial residues. (i) Polymer phase and morphology must be co-designed: PCL beads act as high-throughput depots, whereas PHB, especially with HS-KL, provides structural gating and supports *late*-phase quality. (ii) Co-location of lignin and polyphenols in the same domains is crucial for true metering and chemical stabilisation: when HS-KL is restricted to fibres (MatB) or absent (MatD), *late tails* are structurally or chemically compromised; when HS-KL and GP-PP share both PHB and PCL phases (MatC), HS-KL–GP-PP complexes limit swelling, stiffen the network, and protect labile GP-PP classes. (iii) Bead–fibre adhesion is a useful tuning parameter: in MatC, partial detachment of HS-KL+GP-PP PCL beads contributes to a beneficial *mid*-time pulse at T_37_, but excessive loss would erode *late* capacity. Nevertheless, loss of GP-PP-loaded beads can provide an additional pathway for cargo release, ensuring further delivery in porous media. (iv) GP-PP-only fibres should not be the main carriers, as they swell excessively at elevated temperatures and collapse porosity, giving poor control (MatD).

Overall, this work shows that PHB/PCL electrospun ± electrosprayed biohybrid scaffolds incorporating lignin and polyphenols from agro-industrial waste can be rationally engineered to deliver CRF-like, temperature-responsive polyphenol release over agronomically relevant time windows. Among the architectures tested, the HS-KL–GP-PP co-located design of MatC emerges as a particularly promising platform for controlled delivery of antioxidant, signalling or biostimulant polyphenols in soils and rhizospheres, and the mechanistic understanding gained here—linking polymer phase, lignin placement, morphology and temperature to *burst–mid–late* behaviour—provides a transferable basis for future CRP systems, including those loaded with other bioactives and evaluated under field conditions.

## Figures and Tables

**Table 1:** Description of the electrospun ± electrosprayed scaffolds in terms of composition, weight percentage of components in solution, deposition mode (electrospinning/electrospraying), and processing conditions.

Scaffolds	Description	Ratio (*w*/*w*)	Rate and Voltage *	ES/ESP	Polymers (%_Tot_ *w*/*w*)	Lignin (%_Tot_ *w*/*w*)	Polyphenols (%_Tot_ *w*/*w*)
**MetA**	Combination of		−4.5 KV; (1) 7.3 KV; (2) 8.3 KV				
	-Sol.1) PHB+HS-KL	1:0.033	500 µL/h	ES	64.77% PHB	2.22%	
	-Sol.2) PHB+GP-PP	1:0.088	1600 µL/h	ESP	30.23% PHB		2.78%
**MetB**	Combination of		−10 KV; (1) 8.5 KV; (7) 14 KV				
	-Sol.1) PHB+HS-KL	1:0.033	500 µL/h	ES	61.75% PHB	2.02%	
	-Sol.7) PCL+GP-PP	1:0.075	1600 µL/h	ESP	33.70% PCL		2.53%
**MetC**	Combination of		−3.5 KV; (3) 7.3 KV; (4) 9.3 KV				
	-Sol.3) PHB+HS-KL+GP-PP	1:0.065:0.058	750 µL/h	ES	41.36% PHB	2.71%	2.42%
	-Sol.4) PCL+HS-KL+GP-PP	1:0.056:0.050	750 µL/h	ES	48.38% PCL	2.71%	2.42%
**MetD**	Combination of		−5.5 KV; (5) 7.6 KV; (6) 10.3 KV				
	-Sol.5) PHB+GP-PP	1:0.058	750 µL/h	ES	43.73% PHB	0%	2.56%
	-Sol.6) PCL+GP-PP	1:0.050	750 µL/h	ES	51.15% PCL	0%	2.56%

Note: * In the voltage conditions, the first value corresponds to the negative potential applied to the collector, while the second and third values refer to the positive voltage applied to the needle during electrospinning and electrospraying.

**Table 2:** Polyphenol composition of the extract obtained from grape-pomace waste.

Polyphenol Classes	Compounds	Content (mg mL^−1^)	Percent of Total	Percent Within Class
**Anthocyanins**	*Total*	141.28	30.2	—
	Malvidin-3-O-glucoside	40.88	8.7	28.9
	Pelunidin-3-O-glucoside	36.27	7.7	25.7
	Cyanidin-3-O-glucoside	32.68	7.0	23.1
	Delphinidin-3-O-glucoside	31.45	6.7	22.3
**Flavan-3-ols**	*Total*	94.38	20.2	—
	Catechins	33.18	7.1	35.2
	Procyanidin dimer B3	31.67	6.8	33.6
	Procyanidin dimer B1	29.53	6.3	31.3
**Flavonols (quercetins)**	*Total*	116.61	24.9	—
	Quercetin	62.3	13.3	53.4
	Quercetin-3-O-glucoside	49.31	10.5	42.3
	Quercetin-3-O-rhamnoside	5.0	1.1	4.3
**Phenolic acids**	*Total*	53.98	11.5	—
	Hydroxybenzoic acids	35.57	7.6	65.9
	Gallic acid	18.41	3.9	34.1
**Stilbenes**	Resveratrol	62.12	13.3	100.0

**Table 3:** Morphological characterisation of fibres and particles comprising the bio-based nanohybrid scaffolds before and after 14-day soaking at 37 °C in 0.11 M phosphate buffer (pH 7.4) based on scanning electron microscopy (SEM) observations.

Scaffolds	Fibre Diameter_p_ (µm)	Fibre Diameter_s_ (µm)	Fibre Diameter Variation (%)	Particle Roundness_p_	Particle Roundness_s_	Particle Roundness Variation (%)
**MatA**	1.03 ± 0.63	1.36 ± 0.46	32.3%	0.75 ± 0.10	0.80 ± 0.10	6.4%
**MatB**	1.21 ± 0.34	1.42 ± 0.31	17.0%	0.82 ± 0.10	0.82 ± 0.09	0%
**MatC**	0.23 ± 0.06	0.24 ± 0.06	5.1%	0.83 ± 0.08	0.88 ± 0.07	5.3%
**MatD**	0.28 ± 0.07	0.76 ± 0.18	174.4%	0.84 ± 0.09	0.78 ± 0.13	−6.9%
**Scaffolds**	**Particle_p_ area** **(µm^2^)**	**Particle_s_ area** **(µm^2^)**	**Particle area variation (%)**	**Particle_p_ area_Tot_** **(µm^2^)**	**Particle_s_ area_Tot_** **(µm^2^)**	**Particle Area_Tot_ variation** **(%)**
**MatA**	1.03 ± 0.43	1.05 ± 0.34	1.3%	51.7	53.4	3.4%
**MatB**	7.31 ± 2.77	4.70 ± 2.09	−35.7%	365.7	235.2	−35.7%
**MatC**	0.81 ± 0.47	0.81 ± 0.41	0.5%	39.5	30.8	−22.1%
**MatD**	2.83 ± 0.96	2.18 ± 1.07	−22.8%	141.4	111.4	−22.5%
**Notes**	*p* = pristine scaffolds *s* = scaffolds upon soaking					

**Table 4:** Matrix-by-matrix peak areas by GP-PP release profile regions, *total* areas, and t_50_ absolute values and percentage differences as a function of temperature (ambient and 37 °C temperatures). Numbers in brackets are the percentages of variation in the values at T_37_ relative to those at T_A_.

Ambient (TA) Peak Areas and t50						
Matrix	*Burst* Region	*Mid* Region	*Late Tail* Region	*Total* Area	*Late* Fraction	t_50_ (h)
**MatA**	9.43	17.53	14.40	41.36	0.35	148
**MatB**	13.25	23.67	20.19	57.10	0.35	144
**MatC**	11.51	19.34	18.51	49.35	0.38	142
**MatD**	7.25	15.92	11.66	34.83	0.33	140
**37 °C (T_37_) peak areas and t_50_**						
**Matrix**	***Burst*** **region**	***Mid*** **region**	***Late tail*** **region**	***Total*** **area**	***Late*** **fraction**	**t_50_ (h)**
**MatA**	11.81	16.27	8.14	36.21	0.22	118
**MatB**	12.60	17.59	6.77	36.96	0.18	103
**MatC**	12.97	19.79	9.76	42.52	0.23	114
**MatD**	7.51	15.21	7.64	30.36	0.25	125
**Δ** **peak areas (absolute and percentage changes)**						
**Matrix**	**Δ*Burst*** **region**	**Δ*Mid*** **region**	**Δ*Late*** **region**	**Δ*Total*** **area**	**Δ*Late*** **fraction**	**∆ t_50_ (h)**
**MatA**	+2.38 (+25.3%)	−1.27 (−7.2%)	−6.26 (−43.5%)	−5.14 (−12.4%)	−0.12 (−34.5%)	−29.84 (−20.1%)
**MatB**	−0.65 (−4.9%)	−6.08 (−25.7%)	−13.41 (−66.5%)	−20.14 (−35.3%)	−0.17 (−48.1%)	−40.50 (−28.2%)
**MatC**	+1.47 (+12.8)	+0.45 (+2.4%)	−8.75 (−47.3%)	−6.83 (−13.8%)	−0.15 (−40.0%)	−28.00 (−19.7%)
**MatD**	+0.26 (+3.5%)	−0.71 (−4.4%)	−4.02 (−34%)	−4.47 (−12.8%)	−0.08 (−23.9%)	−14.88 (−10.7%)
**Causes of variations**						
**Notes**	Rises at 37 °C for MatA and MatC (faster hydration/solubility; in MatC, there is weaker bead–fibre adhesion as well), roughly flat/slightly down for MatB, small uptick for MatD	Largest drop in MatB (PCL bead shrinkage + narrowed pores); MatC is slightly up due to the detached-bead pulse; MatA/MatD down modestly	All down at 37 °C; the biggest fall is MatB, then MatC, MatA, MatD	All down at 37 °C; the biggest fall is MatB, then MatC, MatA, MatD	Earlier at 37 °C for every matrix (the largest shift in MatB, meaning that whatever can leave tends to do so before tightening/ageing fully develops).	Earlier at 37 °C for every matrix (the largest shift in MatB, meaning that whatever can leave tends to do so before tightening/ageing fully develops).

## Data Availability

The data presented in this study are available from the corresponding author upon request. The data are currently stored in IRIS, the institutional open-access repository of the National Research Council of Italy (CNR).

## Supplementary Materials

The following supporting information can be downloaded at: https://www.mdpi.com/article/10.3390/polym18060715/s1. §S1. Nanomaterials: categories and applications; §S2. Agro-industrial waste: constituents and value-added products; §S3. Polyphenols: physicochemical properties and components in the GP extract; §S4. Simulation of a potential polyphenol extract spectrum; §S5. CRF release profiles; §S6. Assessing polyphenol release profiles from matrices at 37 °C, in addition to ambient temperature; §S7. Matrix-by-matrix properties at ambient and 37 °C temperatures; §S8. Potential applications of the biohybrid nanostructures in agriculture. Table S1. List of comprehensive classes of nanomaterials, their general use, some specific applications and final products or processes, as well as the mode of applications. Table S2. List of common polymer nanofibre types, their general uses, some specific applications with final products or processes, and mode of application. Table S3. Common constituent groups, components, and main sources of agro-industrial waste originating from the processing of plant- or animal-derived materials. Table S4. List of main value-added products obtained from agro-industrial waste. Table S5. Some physicochemical properties of polyphenol classes and subclasses. Table S6. Matrix-by-matrix composition and performances at ambient temperature (TA). Table S7. Matrix-by-matrix composition and performances at 37 °C (T37). Table S8. Matrix-by-matrix match between scaffold type (MatA–MatD), temperature scenario (ambient vs warm soils), crop category and management context (e.g., transplant vs direct sowing, protected vs open field), preferred application mode and depth, and the rationale for each choice in terms of *burst*/*mid*/*late* release characteristics. Figure S1. Identification of polyphenol components in the grape-pomace extract through UV-Vis spectra at the respective absorbance wavelengths of aliquots resolved by HPLC: (A) phenolic acids and flavan-3-ols at 280 nm, (B) anthocyanins at 520 nm, (C) stilbenes at 307 nm, and (D) flavonols (quercetins) at 365 nm. Figure S2. Simulated UV-Vis absorbance spectra, in the range 200 nm–800 nm, of some polyphenol classes present in typical plant extracts (flavonoids, phenolic acids, stilbenes, hydrolysable tannins, and lignans), highlighting the absorbance peaks characteristics of each class and of the resulting combination (Total) (not quantitative). The spectra shown here are specifically addressed to the 250–600 nm interval to emphasise diagnostic polyphenol bands; deep-UV absorption (<230 nm), which is typically dominated by strong aromatic π→π* transitions and background contributions, is omitted here. Figure S3. Simulated UV-Vis absorbance spectra, in the range 200 nm–800 nm, of polyphenols in plant extracts exhibiting the dependence on the solvent: water (pH 7.0 buffer) (blue), methanol (orange), ethanol (green), and DMSO (red) (A). Simulated UV-Vis absorbance spectra, in the range 200 nm–800 nm, of polyphenols in plant extracts displaying the dependence on pH: pH 1.0 (blue), 3.5 (orange), 7.4 (green), 9.0 (red), and 11.0 (violet) (B). Simulated UV-Vis absorbance spectra, in the range 150 nm–300 nm, of the various organic components typically present in the grape-pomace extracts upon polyphenol extraction (C). Simulated UV-Vis absorbance spectra, in the range 200 nm–800 nm, of the various polyphenols identified in this study in the grape-pomace (GP) extract and the relative assignment of the absorbance peaks as follows: anthocyanins, flavan-3-ols, flavonols (quercetin family), phenolic acids, and stilbenes (D). The UV-Vis absorbance spectrum in D considers the solvent used for the GP extract (methanol:water, 80:20 *v*/*v*) and the phosphate buffer (pH 7.4), where the extract absorbance was measured. Peaks modelled as Gaussian; total is normalised to a maximum of 1 for display (i.e., considering only the compounds measured and not including any unquantified polyphenols). A, B, and C are not quantitative. D refers to the total amount measured (Table 2) and accounts for the fact that the visible anthocyanin peak fades as pH increases, becoming progressively colourless. The spectra shown here are specifically addressed to the 250–600 nm interval to emphasise diagnostic polyphenol bands; deep-UV absorption (<230 nm), which is typically dominated by strong aromatic π→π* transitions and background contributions, is omitted here. Figure S4. Schematic of typical CRF release rate profiles with burst, mid, and late tail phases, as typical of monomodal, as well as bimodal and multimodal CRF.

## Abbreviations

The following abbreviations are used in this manuscript:

PHB	Poly-3-hydroxybutyric acid
PCL	Polycaprolactone
CRFs	Controlled-release fertilisers
SRFs	Slow-release fertilisers
CRPs	Controlled-release polyphenols
HSs	Hazelnut shells
HS-KL	Hazelnut-shell Klason lignin (acid-insoluble fraction by Klason method); not Kraft lignin
GP	Grape pomace
GP-PPs	Grape-pomace polyphenols
(FE-)SEM	Field-emission scanning electron microscopy
FTIR-ATR	Fourier transform infrared–attenuated total reflectance
UV–VIS	Ultraviolet–visible radiation
WCA	Water contact angle
WDV	Water droplet volume
RAF	Rigid amorphous fraction
MAF	Mobile amorphous fraction
HBA	Hydroxybenzoic acid
