# Supplementary material for: Agro-Industrial Biowaste Valorisation by Engineering Controlled-Release Polyphenol Products for Applications in Sustainable Agriculture
Source: Polymers (Basel). 2026 Mar 16;18(6):715. doi: 10.3390/polym18060715 (PMC13029903; doi:10.3390/polym18060715)
Supplement: Supplementary file 1 [file polymers-18-00715-s001.zip › polymers-4175207-supplementary.pdf]

Supplementary Materials

Engineering Bio-based Controlled Release Polyphenol Products for Applications in Sustainable Agriculture

Fabrizio De Cesare [1,2,*], Simone Serrecchia [2], Gabriella Di Carlo [4], Cristina Riccucci [4], Gianmarco Alfieri[1], Andrea Bellincontro[1], Sarai Agustin Salazar [3], Gabriella Santagata [3], Paolo Papa [2], Antonella Macagnano [2,*]

[1] Department for Innovation in Biological, AgriFood and Forest Systems (DIBAF), University of Tuscia, Viterbo (VT), Italy; decesare@unitus.it (F.D.C.); gian.alfieri@unitus.it (G.A.); bellin@unitus.it (A.B.)

[2] Institute of Atmospheric Pollution Research (IIA), National Research Council (CNR), Montelibretti (RM), Italy; simoneserrecchia@cnr.it (S.S.); antonella.macagnano@cnr.it (A.M.); paolo.papa@cnr.it (P.P.); decesare@unitus.it (F.D.C.)

[3] Institute of Polymers, Composites and Biomaterials (IPCB), National Research Council (CNR), Pozzuoli (NA), Italy; sarai.agustinsalazar@cnr.it (S.A.S.); gabriella.santagata@ipcb.cnr.it (G.S.)

[4] Institute for the Study of Nanostructured Materials (ISMN), National Research Council (CNR), Montelibretti (RM), Italy; gabriella.dicarlo@cnr.it (G.D.C.); cristina.riccucci@cnr.it (C.R.)

* Correspondence: antonella.macagnano@cnr.it (A.M.); decesare@unitus.it (F.D.C.)

Table of Contents

Table S1. List of comprehensive classes of nanomaterials, their general use, some specific applications and final products or processes, and mode of applications.

Table S2. List of common polymer nanofibre types, their general uses, some specific applications with final products or processes, and mode of application.

Table S3. Common constituent groups, components and main sources of agro-industrial waste originating from the processing of plant- or animal-derived materials.

Table S4. List of main value-added products obtained from agro-industrial waste.

Table S5. Some physicochemical properties of polyphenol classes and subclasses.

Table S6. Matrix-by-matrix composition and performances at ambient temperature (T_A).

Table S7. Matrix-by-matrix composition and performances at 37 °C (T_{37}).

Table S8. Matrix-by-matrix match between scaffold type (MatA–MatD), temperature scenario (ambient vs warm soils), crop category and management context (e.g., transplant vs direct sowing, protected vs open field), preferred application mode and depth, and the rationale for each choice in terms of burst/mid/late release characteristics.

Figure S1. Identification of polyphenol components in the grape pomace extract through UV–Vis spectra at the respective absorbance wavelengths of aliquots resolved by HPLC: A) phenolic acids and flavan-3-ols at 280 nm, B) anthocyanins at 520 nm, C) stilbenes at 307 nm, and D) flavonols (quercetins) at 365 nm.

Figure S2. Simulated UV–Vis absorbance spectra, in the range 200 nm-800 nm, of some polyphenol classes present in typical plant extracts (flavonoids, phenolic acids, stilbenes, hydrolysable tannins, and lignans), highlighting the absorbance peaks characteristics of each class and of the resulting combination (Total) (not quantitative). The spectra shown here are specifically addressed to the 250–600 nm interval to emphasise diagnostic polyphenol bands; deep-UV absorption (<230 nm), which is typically dominated by strong aromatic $\pi \rightarrow \pi^*$ transitions and background contributions, is here omitted.

Figure S3. Simulated UV–Vis absorbance spectra, in the range 200 nm-800 nm, of polyphenols in plant extracts exhibiting the dependence on the solvent: water (pH 7.0 buffer) (blue), methanol (orange), ethanol (green), and DMSO (red) (A). Simulated UV–Vis absorbance spectra, in the range 200 nm-800 nm, of polyphenols in plant extracts displaying the dependence on pH: pH 1.0 (blue), 3.5 (orange), 7.4 (green), 9.0 (red), and 11.0 (violet) (B). Simulated UV–Vis absorbance spectra, in the range 150 nm-300 nm, of the various organic components typically present in the grape-pomace extracts upon polyphenol extraction (C). Simulated UV–Vis absorbance spectra, in the range 200 nm-800 nm, of the various polyphenols identified in this study in the grape-pomace (GP) extract and the relative assignment of the absorbance peaks as follows: anthocyanins, flavan-3-ols, flavonols (quercetin family), phenolic acids, and stilbenes (D). The UV–Vis absorbance spectrum in D accounts for the solvents used for the GP extract (methanol:water, 80:20 v/v) and the phosphate buffer at pH 7.4, in which the extract absorbance was measured. Peaks modelled as Gaussians; total is normalised to a maximum of 1 for display (i.e. considering only the compounds measured and not including any unquantified polyphenols). A, B, and C are not quantitative. D refers to the total amount measured (Table 2) and accounts for the fading of the visible anthocyanin peak as pH increases, resulting in progressive colourlessness. The spectra shown here are limited to the 250−600 nm interval to emphasise diagnostic polyphenol bands; deep-UV absorption (<230 nm), which is typically dominated by strong aromatic $\pi \rightarrow \pi^*$ transitions and background contributions, is omitted here.

Figure S4. Schematic of typical CRF release rate profiles with burst, mid and late tail phases as typical of monomodal, bimodal and multimodal CRF.

§S1. Nanomaterials: categories and applications.

§S2. Agro-industrial waste: constituents and value-added products.

§S3. Polyphenols: physicochemical properties and components in the GP extract.

§S4. Simulation of a potential polyphenol extract spectrum.

§S5. CRF release profiles.

§S6. Assessing polyphenol release profiles from matrices at 37 °C, in addition to ambient temperature.

§S7. Matrix-by-matrix properties at ambient and 37 °C temperatures.

§S8. Potential applications of the biohybrid nanostructures in agriculture.

References

§S1. Nanomaterials: categories and applications

TABLE S1. List of comprehensive classes of nanomaterials, their general use, some specific applications and final products or processes, and mode of applications.

Nanomaterial Class	General Use Category	Specific Applications	Products/Processes	Main Mode of Application
**Carbon Nanotubes (CNTs)**	Electrical conductivity, reinforcement	Flexible electronics, sensors, reinforced composites	Conductive films, biosensors	Incorporated in polymers or inks
**Graphene and Graphene Oxide**	Thermal/electrical conductivity, barrier enhancement	Batteries, membranes, coatings, biosensors	Supercapacitors, water filtration membranes	Surface coatings, films, composites
**Quantum Dots**	Fluorescence, biosensing	Bioimaging, quantum computing, medical diagnostics	Quantum dot TVs, cancer imaging agents	Suspended in colloidal systems
**Metallic Nanoparticles (Ag, Au, Cu)**	Antimicrobial, catalytic	Wound healing, tissue regeneration, catalysis	Silver-coated bandages, gold nanosensors	Dispersed in polymers, coated on surfaces
**Metal Oxide Nanoparticles (TiO_2, ZnO, Fe_3O_4)**	Photocatalysis, magnetic, antibacterial	Environmental remediation, self-cleaning surfaces	Sunscreens, water treatment	Integrated into matrices or aqueous dispersions
**Nanoclays**	Barrier improvement, controlled release	Food packaging	Edible coatings, films	Blended in packaging
**Silica Nanoparticles (SiO_2)**	Surface area and carrier matrix	Drug delivery	Vaccine carriers	Surface treatment or matrix incorporation
**Liposomes**	Drug delivery, encapsulation	Pharmaceuticals, nutraceuticals	Liposome-based drug carriers	Nanoemulsions, injection
**Polymeric Nanoparticles**	Controlled delivery, biodegradability	Biodegradable drug release systems, vaccines	PLGA nanoparticles in medicine	Encapsulated and released in fluids
**Dendrimers**	Targeted drug delivery, gene therapy	Cancer therapy, diagnostic carriers	Dendrimer-based gene delivery systems	Covalent bonding to payloads

Table S2. List of common polymer nanofibre types, their general uses, some specific applications with final products or processes, and mode of application.

Polymer Nanofibres	General Use Category	Specific Applications	Products/Processes	Main Mode of Application
**Poly(lactic acid) (PLA) Nanofibres**	Biodegradable scaffolds, packaging, environmental sensing, agriculture	Biodegradable filters, VOC sensors, food packaging	PLA filters for VOC capture	Electrospun membranes, sensor films, biodegradable agro-films
**Polycaprolactone (PCL) Nanofibres**	Tissue engineering, drug delivery, pollution detection, agriculture	Implant coatings, pollutant-detecting mats, controlled fertilizer release	PCL mats for drug and nutrient release	Mats with embedded sensing or releasing agents
**Poly(vinyl alcohol) (PVA) Nanofibres**	Wound dressing, biosensing, water filtration, agriculture	Water purification membranes, pathogen-detecting sensors, plant stress biosensors	PVA-CNT plant biosensors, water control membranes	Blended nanofibre sheets for sensors or release systems
**Polyurethane (PU) Nanofibres**	Elastic biomaterials, filtration, sensor substrates, agriculture	Dust filters, crop-protecting textiles	PU covers for field sensors, filter layers	Nanotextile barriers or protective veils
**Polyacrylonitrile (PAN) Nanofibres**	Carbon precursor, filtration, energy storage, agriculture	Li-ion battery electrodes, CO_2 scrubbers, nanofibrous mulch materials	PAN mulch films, battery-supported irrigation	Carbonized fibres in energy devices or soil amendments
**Poly(lactic-co-glycolic acid) (PLGA) Nanofibres**	Controlled drug delivery, environmental detox systems, agriculture	Detoxification systems, sustained pesticide release	PLGA mats for dual herbicide release	Multilayered mats for gradual agrochemical release
**Gelatin Nanofibres**	Tissue regeneration, biosorbent materials, agriculture	Wound healing and nutrient-retaining scaffolds for roots	Gelatin scaffolds for rhizosphere conditioning	Root-targeted scaffold structures
**Chitosan Nanofibres**	Antimicrobial membranes, water remediation, agriculture	Dye-adsorbing fibres, antimicrobial plant wraps	Chitosan-PVA membranes against fungal infections	Electrospun antimicrobial wraps and soil mats

§S2. Agro-industrial waste: constituents and value-added products

Table S3. Common constituent groups, components and main sources of agro-industrial waste originating from the processing of plant- or animal-derived materials.

Constituent Groups	Typical Components	Main Sources
Organic Matter	Cellulose, hemicellulose, lignin, pectin, starch, proteins, lipids	Bagasse, cereal husks, fruit/vegetable pomace, oilseed cakes
Minerals & Inorganics	Macronutrients (K, Ca, Mg, P); trace elements (Fe, Zn); ash (Si, Na, Cl)	Processing residues, filter dust, spent washing water
Bioactives & Functional Compounds	Polyphenols, flavonoids, essential oils, antioxidants, pigments	Grape, citrus, olive, tea, tomato, apple processing residues

Table S4. List of main value-added products obtained from agro-industrial waste.

Product Category	Derived From	Typical Use Sector	Notable Examples
Biopolymers	Cellulose, hemicellulose, lignin-rich residues	Packaging, biomedical, textile industry	Polylactic acid (PLA), polyhydroxyalkanoates (PHAs)
Bioplastics	Starch-rich or cellulose-based biomass	Biodegradable consumer goods	Starch-based plastic films
Animal Feed	Fruit/vegetable peels, oilseed cakes, brewery residues	Livestock nutrition	Dried fruit pomace pellets
Protein Hydrolysates	Protein-rich residues (e.g. oilseed cakes, dairy waste)	Animal feed, fertilizer additives	Amino acid-rich feed additives
Paper and Pulp	Lignocellulosic waste like straw, bagasse	Packaging, hygiene products	Recycled paper, molded fiber products
Biofuels	Starch/cellulose-based waste (e.g. molasses, husks)	Energy, transport	Bioethanol, biogas
Enzymes	Fruit peels, pomace, bran, spent grains	Food processing, textile, detergents	Cellulase, amylase, pectinase
Nutraceuticals	Fruit and vegetable pomace, citrus peel, grape seeds	Functional foods, dietary supplements	Resveratrol, limonene, flavonoids
Bioactive Compounds	Vegetable residues, herb waste, grape pomace	Agricultural biostimulants, pesticides	Phenolics, alkaloids, saponins

§S3. Polyphenols: physicochemical properties and components in the GP extract

Table S5. Some physicochemical properties of polyphenol classes and subclasses.

Main Class	Subclass	Examples	Hydrophilicity	LogP (Range)	Water Solubility
Phenolic acids	Hydroxybenzoic acids	Gallic acid, Protocatechuic acid	High	0.7-1.1	Moderate–High
Flavonoids	Flavanols (aglycones)	(+/−)-Catechin	Low	0.4	Low
	Flavonols (aglycones)	Quercetin	Low–Moderate*	1.5	Low–Moderate*
	Flavones	Apigenin, Luteolin	Low–Moderate*	2.2–3.1	Low–Moderate*
	Flavanones	Naringenin, Hesperetin	Low–Moderate*	2.0–3.5	Low–Moderate*
	Anthocyanins	Cyanidin-3-glucoside	Very High	0.39-0.98	Low–Moderate
	Isoflavones	Genistein, Daidzein	Low	2.0–3.1	Low
Stilbenes	Chalcones	Phloretin, Butein	Low	2.5–3.5	Low
Lignans		Resveratrol (aglycone)	Low	3.1	Low
Notes	LogP measures the partition of neutral polyphenol molecules at equilibrium between n-octanol and water				

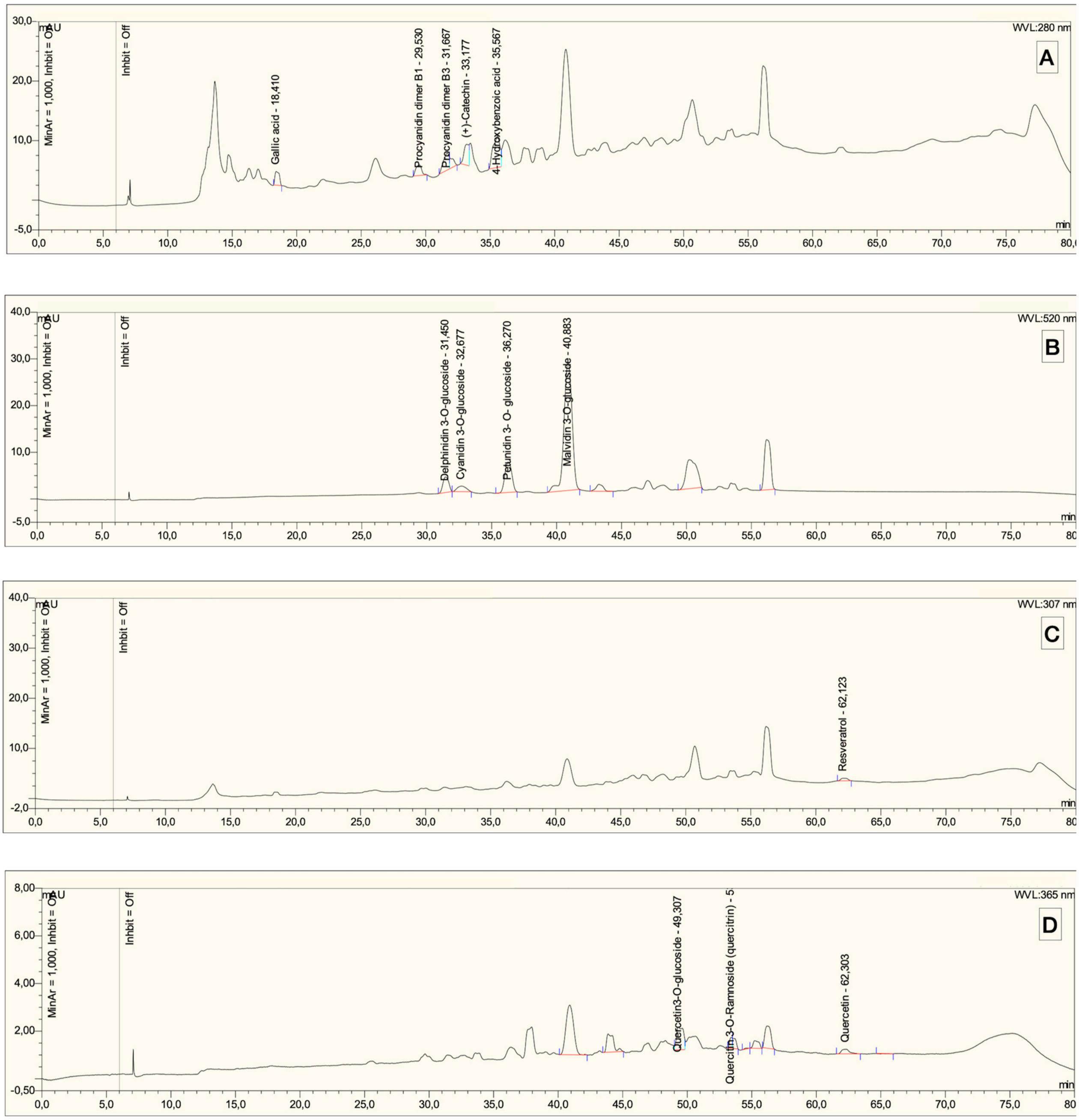

Figure S1. Identification of polyphenol components in the grape pomace extract through UV–Vis spectra at the respective absorbance wavelengths of aliquots resolved by HPLC: A) phenolic acids and flavan-3-ols at 280 nm, B) anthocyanins at 520 nm, C) stilbenes at 307 nm, and D) flavonols (quercetins) at 365 nm.

§S4. Simulation of a potential polyphenol extract spectrum

Polyphenolic extracts are typically composed of a multitude of different polyphenols (flavonoids, phenolic acids, stilbenes, hydrolysable tannins, lignans, etc.) (Figure 1) with varying light absorbances. Typically, polyphenol extracts exhibit a complex UV–Vis absorbance spectrum in the 200-800 nm range, with peaks arising from diverse polyphenol classes (Figure S2).

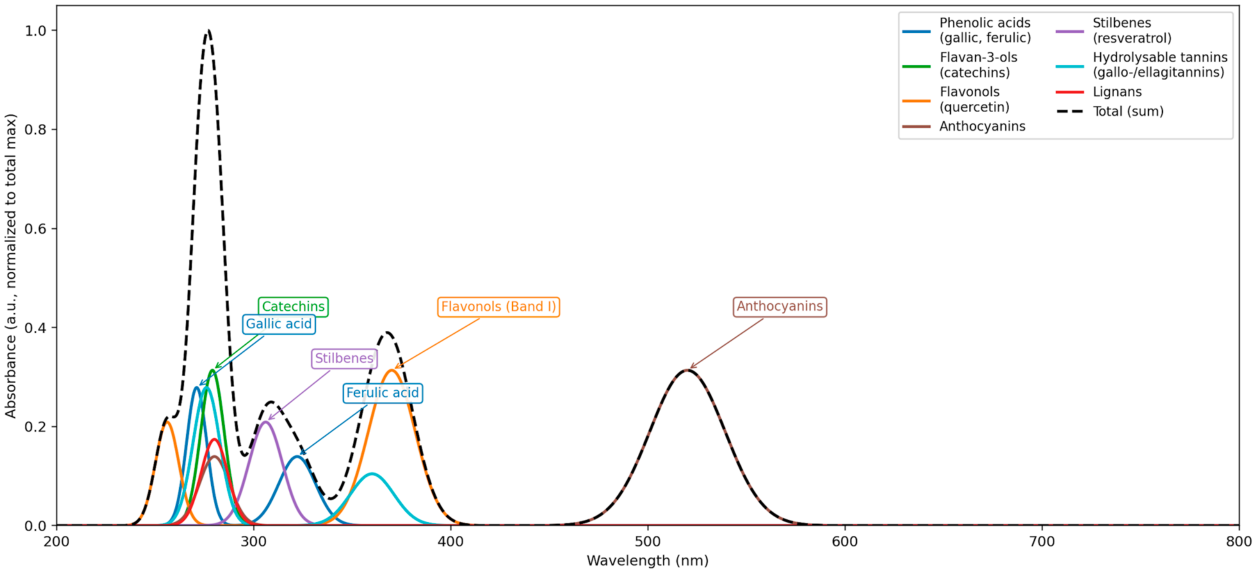

Figure S2. Simulated UV–Vis absorbance spectra, in the range 200 nm-800 nm, of some polyphenol classes present in typical plant extracts (flavonoids, phenolic acids, stilbenes, hydrolysable tannins, and lignans), highlighting the absorbance peaks characteristics of each class and of the resulting combination (Total) (not quantitative). The spectra shown here are specifically addressed to the 250 – 600 nm interval to emphasise diagnostic polyphenol bands; deep-UV absorption (<230 nm), which is typically dominated by strong aromatic $\pi \rightarrow \pi^*$ transitions and background contributions, is here omitted.

As highlighted in Figure S2, the main diagnostic polyphenol peaks observed in the PP extracts can be assigned as follows:

~250–300 nm: II – characteristic of low-energy π–π* transitions (Band II), often described as "benzenoid/benzoyl system" transitions in flavonoids and due to hydroxyl and carbonyl groups within aromatic structures typical of phenolic acids (like gallic acid) and flavan-3-ols.

~320–330 nm: shoulder from hydroxycinnamic acids (e.g., ferulic) and stilbenes.

~350–380 nm: Band I region of flavonols (like quercetin)/flavones - often small unless aglycones are abundant) and hydrolysable tannins (e.g., gallo-/ellegitannins).

~500-540 nm: typically pH-dependent since often associated with specific compounds like anthocyanins; it is typically due to π–π*/charge-transfer-like character.

In this study, the polyphenolic extract was investigated by dissolving 10 µl of the methanol:water (80:20 v/v) extract in 990 µl of phosphate 0.11 M 0.11 M phosphate buffer (pH 7.4) at 37 °C (pH 7.4) at 37 °C (0.11 M, pH 7.4) and measuring its UV–Vis absorbance spectrum from 195 nm to 700 nm. Indeed, Figure 3a shows that the absorbance spectra of the various compounds extracted with the methanol:water (80:20 v/v) solution exhibited two peaks, designated Peak 1 and Peak 2, with maxima at 208 nm and 280 nm, respectively, and a shoulder at 320-330 nm. Hence, the PP-extract spectrum observed here is substantially consistent with those reported in the literature, although with some differences. These divergences can be attributed to the solvent used for spectroscopic analysis, which is known to affect the recorded spectrum [1] (Figure S3A).

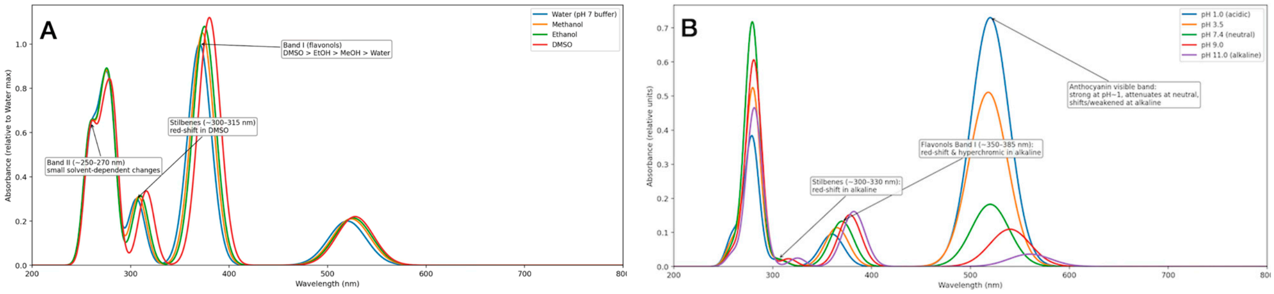

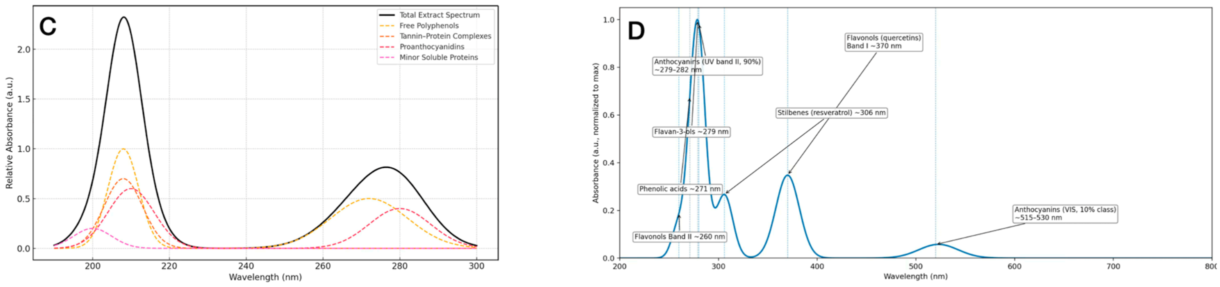

Figure S3. Simulated UV–Vis absorbance spectra, in the range 200 nm-800 nm, of polyphenols in plant extracts exhibiting the dependence on the solvent: water (pH 7.0 buffer) (blue), methanol (orange), ethanol (green), and DMSO (red) (A). Simulated UV–Vis absorbance spectra, in the range 200 nm-800 nm, of polyphenols in plant extracts displaying the dependence on pH: pH 1.0 (blue), 3.5 (orange), 7.4 (green), 9.0 (red), and 11.0 (violet) (B). Simulated UV–Vis absorbance spectra, in the range 150 nm-300 nm, of the various organic components typically present in the grape-pomace extracts upon polyphenol extraction (C). Simulated UV–Vis absorbance spectra, in the range 200 nm-800 nm, of the various polyphenols identified in this study in the grape-pomace (GP) extract and the relative assignment of the absorbance peaks as follows: anthocyanins, flavan-3-ols, flavonols (quercetin family), phenolic acids, and stilbenes (D). The UV–Vis absorbance spectrum in D considers the solvent used for the GP extract (methanol:water, 80:20 v/v) and the phosphate buffer at pH 7.4, in which the extract absorbance was measured. Peaks modelled as Gaussians; total is normalised to a maximum of 1 for display (i.e. considering only the compounds measured and not including any unquantified polyphenols). A, B, and C are not quantitative. D refers to the total amount measured (Table 2) and accounts for the fact that the visible anthocyanin peak fades with increasing pH, becoming progressively colourless. The spectra shown here are specifically addressed to the 250 – 600 nm interval to emphasise diagnostic polyphenol bands; deep-UV absorption (<230 nm), which is typically dominated by strong aromatic $\pi \rightarrow \pi^*$ transitions and background contributions, is here omitted.

The pH of a solution also significantly affects the UV–Vis absorbance spectra of polyphenols because protonation and deprotonation of their phenolic hydroxyl groups alter their electronic structure and conjugation [2] . This causes shifts of the maximum absorption wavelength (λ_{max}) and changes in absorption intensity, as reported in Figure S3B, which simulates this effect in the case of the classes of polyphenols identified in this study: i) anthocyanins: strong visible band near ~520 nm at pH≈1, which progressively attenuates at neutral, and weak, red-shifted at alkaline; ii) flavonols (Band I ~350–385 nm): red-shift + hyperchromic with alkalinity (deprotonation); iii) stilbenes (~300–330 nm): modest red-shift and intensity gain in alkaline media; iv) Band II (~250–270 nm; aromatic-ring π–π*): only small pH dependence across classes.

It is worth noting that the methanol:water (80:20 v/v) solution used for polyphenol extraction from grape pomace can, however, also extract other organic substances from grape pomace cells upon lysis. Hence, the typical absorbance spectrum of whole extracts from grape pomace in the range of 190 nm to 700 nm will reflect the combined absorbance of multiple compounds, with possible overlaps. This possibility was assessed by reproducing Gaussian models to simulate the UV spectra of the individual components typically found in grape-pomace extracts: free polyphenols, protein-bound polyphenols, proanthocyanidins, and minor proteins (Figure S3C). Absorption peaks were selected based on known literature data [3–6] . Figure S3D displays a simulated UV–Vis absorbance spectra, in the range 200 nm-800 nm, of the grape-pomace (GP) extract obtained in this study, where the absorbance peaks of the various polyphenols here identified are exhibited and relatively assigned as follows: anthocyanins, split to 10% visible (~520 nm) and 90% to UV Band II (~280 nm); flavonols, split to 70% Band I (~370 nm) and to 30% Band II (~260 nm), consistent with deprotonation enhancing Band I; flavan-3-ols, single UV band ~279 nm; phenolic acids, UV band ~271 nm; and stilbenes (resveratrol), UV band ~306 nm. The UV–Vis absorbance spectrum reported here accounts for the solvent used for the GP extract (methanol:water, 80:20 v/v) and the phosphate buffer at pH 7.4, in which the extract absorbance was measured. Peaks modelled as Gaussians; total normalised to a maximum of 1 for display (i.e. considering only the compounds measured and not including any unquantified polyphenols). A, B, and C are not quantitative. D refers to the total amount measured (Table 2) and accounts for the fact that the visible anthocyanin peak fades with increasing pH,

becoming progressively colourless [7,8] . Compared with Figure S1, the simulated UV absorbance spectrum of a general polyphenol extract from a GP extract reproduces the typical spectrum of polyphenol extracts from plants.

As a consequence, the spectroscopic measurements generally used to evaluate the quantitative release of specific compounds from matrices into incubation solutions, by detecting absorbance at optimal wavelengths and at definite incubation times relative to a reference curve, would rarely have succeeded in identifying the presence of a single polyphenol and quantifying its release. In addition, relative to the absorbance spectrum of the PP extract at T_0, resulting, as said, from the combination of several absorbance spectra, not all the compounds will be presumably released contemporarily from a matrix, because of different interactions and bindings with the carrier polymers. Hence, the polyphenol release at a particular incubation time could involve only some compounds with a slightly different maximum absorbance wavelength than that of the whole extract at T_0. Consequently, using a single maximum absorbance wavelength (that of the whole extract at T_0) for all sampling times may lead to incorrect measurements (over- or underestimation).

To prevent such incorrect estimates, the release of polyphenols from the nanofabrics was quantified by recording the UV–Vis absorbance spectrum of the soaking solution at specified time points, as follows. The absorbance spectrum of the soaking solution in the range of 190 nm to 700 nm was measured for each nanostructured mat on each day of incubation, soon after nanoframework removal, and the observed absorbance peak areas were recorded. The peak areas were then integrated and calculated using UVProbe 2.50 software (Shimadzu UV–Vis Spectrophotometers). Soon after the spectroscopic measurement, the buffer solution was then discarded and replaced with a fresh one in which the scaffold was immersed again. This procedure was repeated daily for two weeks for each nanofabric type.

Although unusual, this approach is more reliable than the one typically used (i.e. measuring the absorbance at a single wavelength of a solution where a matrix is permanently soaked throughout a fixed period) for two reasons: i) that one described above about a single wavelength (e.g. underestimation), ii) the dynamics of the molecule release by diffusion from matrices as well as organisms (e.g. plant roots) depend on the concentration gradient of that molecule (described by the Fick s Law) between inside the matrix/organism and the outer environment. At T_0, the concentration gradient is high, and diffusion is rapid; however, it decreases over time as the concentration of molecules in the external solution increases. Incidentally, these dynamics are very rare in natural conditions, e.g., for growing plants in both hydroponics and soil, since molecules released into the external medium rarely accumulate. On the contrary, they rapidly decrease in concentration due to various processes (absorption by organisms, adsorption onto substrates, discharge, leaching, runoff, etc.). Hence, daily renewal of the soaking solution maintains a high gradient, allowing for more homogeneous and comparable polyphenol-release measurements over time, as measurements are conducted under similar conditions.

§S5. CRF release profiles

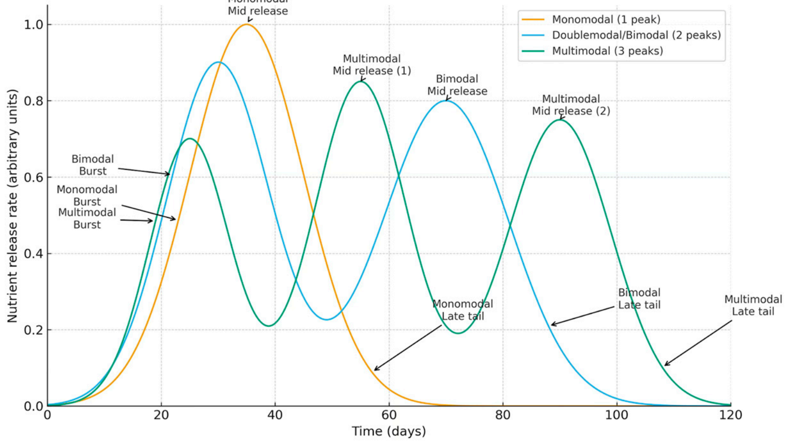

Figure S4. Schematic of typical CRF release rate profiles with *burst*, *mid* and *late tail* phases as typical of monomodal, bimodal and multimodal CRF.

§S6. Assessing polyphenol release profiles from matrices at 37 °C, in addition to ambient temperature

The diverse scaffolds created here were tested at two temperatures: ambient (~25°C) and 37°C. Soil temperature typically fluctuates between ~10 and 30 °C [9,10] . Hence, ambient-temperature rankings are most relevant for soil deployment of the proposed delivery matrices, since 37 °C is higher than the average growing-season soil temperature in most environments, such a temperature is realistic because it is also observed under natural and anthropogenic conditions. Indeed, soil temperatures of 37 °C are very plausible at shallow depths (0–5 cm, sometimes 10 cm) in many settings worldwide, during hot spells. These conditions are most common when air T_{max} ≥ 35 °C, soils are dry, sparsely vegetated, dark/tilled or plastic-mulched, south-facing, or recently burned [9,11] . Hot, arid and semi-arid (deserts, drylands): midday near-surface soil temperatures often exceed 35–40 °C in summer, especially under clear skies and low soil moisture [12] . Moreover, in tropical/subtropical croplands, plastic mulches or bare tilled soil can raise soil temperatures to 37–40 °C at a few cm depth on hot days [13] . Mediterranean regions (Spain, Italy, Greece and coastal North Africa) regularly experience such conditions. Here, in drylands and grasslands, recurrent heatwaves and summer dryness frequently drive air temperatures > 35 °C, pushing shallow soils above 35-37 °C on hot days and in open/bare/tilled areas] [14] . In addition, using plastic mulch (clear/black) in these regions raises soil temperature; near-surface values > 40 °C have been reported under extreme heat, making 37 °C routine at midday [15]. Post-fire vegetation loss removes canopy and ground cover, substantially raising soil surface temperatures; analyses of Greek wildfires showed marked soil-level warming after the 2024 fires [17]. Soil depth considerations indicate that the 0–5 cm layer is most responsive, and soil temperatures >37 °C are common in the upper layers due to radiative heating [9] . On the contrary, vegetation cover (tree canopy/cover crops) reduces soil maxima and daily amplitude; shading and moisture buffering can keep the same soil several °C cooler [16] . Satellite and reanalysis summaries for 2017–2023 documented exceptional hot summers across the Mediterranean basin, making soil temperatures ≥ 37 °C at 0–5 cm entirely expected [17] . Field studies in Mediterranean agricultural lands (vineyards/orchards) showed that inter-row bare soil and plastic mulches (commonly used to warm soils) can push shallow soil temperatures to 37–40 °C at midday during heat events [14] . Hence, 37 °C was used as a stress test to rank the scaffolds.

Temperature can affect not only soil ecosystems and processes but also polymeric delivery systems and was therefore used in this study as a stress test to assess the performance of the matrices created here.

§S7. Matrix-by-matrix properties at ambient and 37 °C temperatures

Table S6. Matrix-by-matrix composition and performances at ambient temperature (T_A)

Matrix	Composition (who holds what)	Key structural domains (as-made)	MAF/RAF × HS-KL × polyphenols (interplay)	Dominant diffusion mechanisms	Release outcomes (*burst / mid / late*)†	Net conclusions (pros & cons)
**MatA**	Fibres: PHB + HS-KL (no GP-PP). Beads: PHB + GP-PP (no HS-KL). (No co-location.)	PHB fibres:higher crystalline lamellae + RAF; modest MAF continuity. HS-KL in fibres increases surface polarity. PHB beads: semi-crystalline depots; depot geometry set by ambient bead area & count.	In beads, GP-PP sit in PHB MAF pockets separated by lamellae/RAF → slow in-depot diffusion. No π–π/H-bond metering (HS-KL absent in depots). HS-KL in fibres raises wettability but does not meter depots.	Network-limited + depot-gated: baseline porosity/tortuosity from fibre diameter/packing throttles transport; PHB depots further gate diffusion via RAF.	*Burst*: weak (PHB depots). *Mid*: modest. *Late tail:* present, modest; flavonols/stilbenes dominate later. *Total*: Low–Medium. *Late fraction:* Low–Medium (3rd) (MatB > MatC > MatA > MatD). t_{50} = the highest	Pro: Chemically simple; stable scaffold. Con: Lacks co-located HS-KL → little metering; PHB depots slow → lower *total* and later t_{50}. Best when a very restrained release is desired.
**MatB**	Fibres: PHB + HS-KL. Beads: PCL + GP-PP (no HS-KL). (No co-location.)	PHB fibres: set pore network (as-made fibre diam/packing). PCL beads: MAF-rich depots at ambient → readily accessible.	In PCL beads, GP-PP percolate via MAF (fast). No HS-KL in depots → little chemical retention; anthocyanins/HBAs exit early; flavonols/stilbenes sustain *mid/late* flow	Depot-driven throughput: early/*mid* flux dominated by PCL MAF; fabric permeability still matters but is not rate-limiting initially.	*Burst*: highest (surface-proximate in PCL). *Mid*: strongest. *Late tail:* largest of all matrices, though it tends to taper in slope. *Total*: Highest. *Late fraction:* 1st (MatB > MatC > MatA > MatD). t_{50} = second	Pro: Highest *total* + largest *late tail*; strong mid. Con: No HS-KL metering → less control; anthocyanin stability lower than MatC. Excellent when high dose + extended presence are desired.
**MatC**	Fibres: PHB and PCL, each with HS-KL + GP-PP (deposited separately). Beads: PHB and PCL, each with HS-KL + GP-PP (deposited separately). (Co-location in both phases.)	Parallel depots: PCL (fast MAF) + PHB (gated/lamellar). HS-KL co-located near cargo creates hydrated microdomains around the payload. Ambient bead/fibre metrics define the reservoir & pore network.	HS-KL co-located gives π–π/H-bond metering and antioxidant/co-pigmentation stabilisation (anthocyanins most). In PCL domains HS-KL helps keep MAF hydrated; in PHB domains HS-KL near cargo maintains small accessible MAF pockets → smoother, sustained release.	Parallel-depot kinetics + HS-KL-metered desorption: early/*mid* aided by PCL depots; sustained late from HS-KL-metered depots (PHB and remaining PCL).	*Burst*: tempered (due to HS-KL). *Mid:* clear surge, from fast PCL depots running in parallel. *Late tail*: second-largest, smoothest; anthocyanins persist longer vs B. *Total*: High (≈MatB). *Late fraction*: 2nd (MatB > MatC > MatA > MatD). t_{50} = third	Pro: High totals with most controlled, smooth *late*; better anthocyanin integrity. Con: More complex build (dual phases; HS-KL distribution). Top pick for prolonged, controlled biological effect.
**MatD**	Fibres: PHB and PCL (no HS-KL). Beads:PHB and PCL + GP-PP only (no HS-KL). (No HS-KL anywhere.)	Pore network and reservoir set purely by as-made fibre diam/packing and bead geometry. No extra hydration from HS-KL.	GP-PP rely solely on intrinsic polymer pathways (PCL MAF vs PHB RAF/lamellae). No metering/stabilisation: anthocyanins least protected, *mid/late* rely on polymer alone.	Access-limited: through-fabric wetting/permeability and depot interfaces control flux; without HS-KL, effective transport and chemical hold-up are poor.	*Burst*: minimal. *Mid*: weak–modest. *Late tail*: smallest. *Total*: Lowest. *Late fraction*: 4th (MatB > MatC > MatA > MatD). t_{50} = least	Pro: Simplest chemistry. Con: Lowest *total* and weakest sustained phase; no stabilisation or control over profile. Useful only when very low dosing is acceptable.

*† **Burst** = 0–24 h period including Peak 1; **Mid** = 120–144 h, including Peak 2; **Late tail** = 240–264 h plus the terminal shoulder at 336 h including Peak 3; **Total** = the total released polyphenols over 336 h; **Late fraction** = the proportion of the total that occurs in that same late window (Late fraction = Late tail area/Total area); t_{50} = time at which the cumulative RT area reaches 50% (linear interpolation between the timepoints). **Note:** The late tail is a phase, not a single peak, so it legitimately includes both the third main late peak and the final shoulder. Moreover, the **Late tail** number tells how much came late; the **Late fraction** tells how concentrated the profile is in the late phase relative to the matrix's own total (Late/Total)*

Table S7. Matrix-by-matrix composition and performances at 37 °C (T_{37})

Matrix	Composition (who holds what)	Key structural domains (as-made)	MAF/RAF × HS-KL × polyphenols (interplay)	Dominant diffusion mechanisms	Release outcomes (*burst / mid / late*)†	Net conclusions (pros & cons)	Matrix
**A**	Fibres: PHB + HS-KL (no GP-PP). Beads: PHB + GP-PP (no HS-KL). (No co-location.)	PHB+HS-KL fibres swell (pores narrow); PHB beads undergo secondary crystallisation / MAF→RAF	PHB depots: MAF is consumed → RAF↑; HS-KL not co-located with GP-PP, so no strong complexes in depots; early hydration boosts *burst*, then RAF/lamellae + pore narrowing dominate and suppress *Mid/Late*.	*Burst*: hydration-first Fickian (↑ vs RT); *Mid*: Fickian in densifying PHB + depot gate; *Late*: desorption-limited (less left, tighter paths)	*Burst*: modest (PHB depots). *Mid*: modest. *Late tail:* small-tempered. *Total*: small–medium. *Late fraction*: low-modest (3rd) (MatD > MatC > MatA > MatB)). t_{50} = 3rd	*Burst*: anthocyanins + HBAs; *Mid*: mixed (less anthocyanin); *Late tail:* flavonols + stilbenes	Transport-choked after early window; very restrained warm-condition dosing
**B**	Fibres: PHB + HS-KL. Beads: PCL + GP-PP (no HS-KL). (No co-location.)	PHB+HS-KL fibres swell; PCL beads shrink uniformly (volume ↓, no bead loss)	PCL depots: MAF volume ↓ (shrinkage), pathways shorter; no HS-KL → little metering; PHB(HS-KL) fibres hydrate but pore narrowing throttles access. Net: *Mid* and *Late* drop as MAF shrinks without chemical compensation.	*Burst*: PCL-MAF Fickian under narrowed pores; *Mid*: shortened-path depot diffusion; *Late*: depot-exhaustion (no HS-KL)	*Burst*: small–moderate (surface-proximate in PCL). *Mid*: tempered. *Late tail:* lowest of all matrices, smoothest. *Total*: low-tempered (≈A). *Late fraction*: least ((MatD > MatC > MatA > MatB). t_{50} = highest	*Burst*: anthocyanins + HBAs; *Mid*: residual anthocyanins → flavonols; *Late tail:* flavonols + stilbenes (smallest *late*)	Still serviceable, but loses RT dominance
**C**	Fibres: PHB and PCL, each with HS-KL + GP-PP (deposited separately). Beads: PHB and PCL, each with HS-KL + GP-PP (deposited separately). (Co-location in both phases.)	Minor fibre swelling; PCL bead detachment (total bead area ↓, avg size ~const)	HS-KL co-located preserves π–π + multi-H-bond complexes in both PHB fibres and PCL depots, maintaining metering and anthocyanin protection; however, bead loss spends some cargo *Mid* and reduces *Late* mass. MAF/RAF: PCL still MAF-led; PHB fibres age (RAF↑) but HS-KL moderates desorption.	*Burst*: HS-KL-moderated + slightly leakier surfaces (↑ vs RT); *Mid*: parallel depot/fibre diffusion + morphology-triggered release from detached PCL(HS-KL+GP-PP) beads; *Late*: HS-KL-metered from remaining depots/fibres	*Burst*: highest (due to HS-KL). *Mid*: clear surge, highest, from fast PCL depots running in parallel. *Late tail:* largest, smoothest. *Total*: Highest. *Late fraction*: 2nd (MatD > MatC > MatA > MatB). t_{50} = least	*Burst*: anthocyanins + HBAs ↑; *Mid*: broad mix (anthocyanins + quercetin + stilbene traces); *Late tail:* flavonols + stilbenes + HS-KL-stabilised anthocyanins	Most structured warm profile; retains "quality" late species; cost = bead loss (adhesion)
**D**	Fibres: PHB and PCL (no HS-KL). Beads: PHB and PCL + GP-PP only (no HS-KL). (No HS-KL anywhere.)	Very strong fibre swelling → porosity collapse; beads harder to access	No HS-KL; PHB ageing (RAF↑) and massive swelling block MAF pathways; PCL MAF cannot compensate because access is throttled. Classes sort only by size/polarity; little *late* retention.	*Burst*: access-limited Fickian (very low); *Mid/Late*: slow percolation through swollen network; depots under-fed	*Burst*: minimal. *Mid*: weak–lowest. *Late tail:* small-modest. *Total*: Lowest. *Late fraction*: highest (MatD > MatC > MatA > MatB). t_{50} = 2nd	*Burst*: minimal; *Mid*: mainly HBAs; *Late tail:* weak	Poorest at 37 °C; no HS-KL to offset structural penalty

*† **Burst** = 0–48 h period including Peak 1; **Mid** = 48–192 h, including Peak 2; Late tail = >192 h plus the terminal shoulder at 336 h including Peak 3; **Total** = the total released polyphenols over 336 h; **Late fraction** = the proportion of the total that occurs in that same late window (Late fraction = Late tail area/Total area); t_{50} = time at which the cumulative RT area reaches 50% (linear interpolation between the timepoints). **Note:** The late tail is a phase, not a single peak, so it legitimately includes both the third main late peak and the final shoulder. Moreover, the **Late tail** number tells how much came late; the **Late fraction** tells how concentrated the profile is in the late phase relative to the matrix's own total (Late/Total)*

§S8. Potential applications of the biohybrid nanostructures in agriculture

Table S8. Matrix-by-matrix match between scaffold type (MatA–MatD), temperature scenario (ambient vs warm soils), crop category and management context (e.g., transplant vs direct sowing, protected vs open field), preferred application mode and depth, and the rationale for each choice in terms of burst/mid/late release characteristics.

Matrix / Temp	Best fit crops & stages	Why it matches the profile	How to deploy
MatC (KL+PP co-located) Ambient (15–25 °C)	**Solanaceae transplants** (tomato, pepper, aubergine), **cucurbits** (zucchini, melon, watermelon), **strawberry** runners; woody nursery stock (olive, citrus, grape) at pot-up	Day-1 burst softens shock; day-5–8 mid supports root flush; day-10–14 tail sustains antioxidant status. KL co-location preserves anthocyanins/flavonols → steadier "quality" late phase.	Patch/ring at 2–5 cm around root ball at transplant. One mat per plant (veg) or 2–3 per shrub/tree liner. Irrigate normally.
MatC (KL+PP co-located) 32-37°C (heat waves, shallow warm soils)	Same crops during summer plantings or hot greenhouse benches	Still the best balance under heat (highest total & best late among the four). Expect larger day-1 and day-5 pulses; late smaller unless bead adhesion is improved.	As above; if routinely >32 °C in root zone, use an "adhesion-fix" version (compatibilized beads) or stack two smaller mats 3–5 cm apart to smooth the mid pulse.
MatB (PHB+KL fibres + PCL(PP) beads) Ambient (15–25 °C)	**Fast leafy greens**(rocket/arugula, baby-leaf mixes, lettuce), **brassica** plugs post-transplant, **processing tomato** in cool spring	Highest total & big late (absolute) at RT → rapid canopy/fiber build in week-1, with some sustain into week-2. Less chemical metering than C, but great throughput in mild weather.	Thin strip/patch under the plug at set. For baby-leaf, band along the row every 15–20 cm. Avoid in hot, very wet beds (can front-load too much).
MatB 32-37°C	Cooler coastal/greenhouse RT only; **not** ideal in hot open fields	Warmth shrinks PCL depots and narrows pores → mid/late collapse.	If heat is likely, switch to **C** or add a touch of KL into beads in a revised B.
MatA (PHB(KL) fibres + PHB(PP) beads) 32-37°C	**Heat-sensitive herbs** (basil), newly grafted cucurbits/solanaceae; situations where you want **low total dose** under heat	Shows a bigger day-1, then self-brakes (PHB ageing/pore tightening). Good when you want a **gentle, controlled** exposure in hot beds.	Small patch at root shoulder; don't stack. Pairs well with mild fertigation.
MatA Ambient (15–25 °C)	Perennials in nursery moves; slower veg starts	Conservative profile; smaller total than B/C but smooth.	As above.
MatD (no KL) Any temp	**Baseline/control** or **very low-dose** needs; short nursery holds	Lowest totals; weak sustain. Useful as a "minimal intervention" control or when you only want a tiny day-1 nudge.	Only where deliberate low dosing is desired.

References

1. Kaeswurm, J.A.H.; Scharinger, A.; Teipel, J.; Buchweitz, M. Absorption Coefficients of Phenolic Structures in Different Solvents Routinely Used for Experiments. *Molecules* **2021**, *26*, 4656, doi:10.3390/molecules26154656.
2. Velho, P.; Rebelo, C.S.; Macedo, E.A. Extraction of Gallic Acid and Ferulic Acid for Application in Hair Supplements. *Molecules* **2023**, *28*, 2369, doi:10.3390/molecules28052369.
3. Panzella, L.; Napolitano, A. Condensed Tannins, a Viable Solution To Meet the Need for Sustainable and Effective Multifunctionality in Food Packaging: Structure, Sources, and Properties. *J. Agric. Food Chem.* **2022**, *70*, 751–758, doi:10.1021/acs.jafc.1c07229.
4. Boulet, J. -C.; Ducasse, M. -A.; Cheynier, V. Ultraviolet Spectroscopy Study of Phenolic Substances and Other Major Compounds in Red Wines: Relationship between Astringency and the Concentration of Phenolic Substances. *Aust. J. Grape Wine Res.* **2017**, *23*, 193–199, doi:10.1111/ajgw.12265.
5. Grasel, F. dos S.; Ferrão, M.F.; Wolf, C.R. Ultraviolet Spectroscopy and Chemometrics for the Identification of Vegetable Tannins. *Ind. Crop. Prod.* **2016**, *91*, 279–285, doi:10.1016/j.indcrop.2016.07.022.
6. Nakamura, K.; Shirato, M.; Ikai, H.; Kanno, T.; Sasaki, K.; Kohno, M.; Niwano, Y. Photo-Irradiation of Proanthocyanidin as a New Disinfection Technique via Reactive Oxygen Species Formation. *PLoS ONE* **2013**, *8*, e60053, doi:10.1371/journal.pone.0060053.
7. Chua, L.S.; Thong, H.Y.; Soo, J. Effect of pH on the Extraction and Stability of Anthocyanins from Jaboticaba Berries. *Food Chemistry Advances* **2024**, *5*, 100835, doi:10.1016/j.focha.2024.100835.
8. Okello, A.; Owuor, B.O.; Namukobe, J.; Okello, D.; Mwabora, J. Influence of the pH of Anthocyanins on the Efficiency of Dye Sensitized Solar Cells. **2022**, *8*, e09921, doi:10.1016/j.heliyon.2022.e09921.
9. Sanford, R.A.; Chee-Sanford, J.C.; Yang, W.H. Diurnal Temperature Variation in Surface Soils: An Underappreciated Control on Microbial Processes. *Front. Microbiol.* **2024**, *15*, 1423984, doi:10.3389/fmicb.2024.1423984.
10. Cheng, Q.; Zhang, M.; Jin, H.; Ren, Y. Spatiotemporal Variation Characteristics of Hourly Soil Temperature in Different Layers in the Low-Latitude Plateau of China. *Front. Environ. Sci.* **2022**, *10*, 1091985, doi:10.3389/fenvs.2022.1091985.

11. Chen, H.; Huang, S.; Quan, C.; Chen, Z.; Xu, M.; Wei, F.; Tang, D. Effects of Different Colors of Plastic-Film Mulching on Soil Temperature, Yield, and Metabolites in Platostoma Palustre. *Sci. Rep.* **2024**, *14*, 5110, doi:10.1038/s41598-024-55406-w.
12. Hillel, D. Soil Temperature and Heat Flow. In *Introduction to Environmental Soil Physics*; Hillel, D., Ed.; Academic Press: Burlington, 2003; pp. 215–233 ISBN 978-0-12-348655-4; doi:10.1016/b978-012348655-4/50013-7.
13. Gill, S.M.; Pps, G.; Pal, R.K.; Singh, N.; IMD, M. Mulching Effects on Soil Temperature and Yield of Pear [Pyrus Pyrifoila (Burm.) Nakai] in Humid Subtropical Climate of Punjab. *MAUSAM* **2022**, *73*, 941–948, doi:10.54302/mausam.v73i4.3215.
14. Costa, J.M.; Egipto, R.; Aguiar, F.C.; Marques, P.; Nogales, A.; Madeira, M. The Role of Soil Temperature in Mediterranean Vineyards in a Climate Change Context. *Front. Plant Sci.* **2023**, *14*, 1145137, doi:10.3389/fpls.2023.1145137.
15. Sellami, M.H.; Mola, I.D.; Ottaiano, L.; Cozzolino, E.; Piano, L. del; Mori, M. Evaluation of Biodegradable Mulch Films on Melon Production and Quality under Mediterranean Field Conditions. *Agronomy* **2024**, *14*, 2075, doi:10.3390/agronomy14092075.
16. Lozano-Parra, J.; Pulido, M.; Lozano-Fondón, C.; Schnabel, S. How Do Soil Moisture and Vegetation Covers Influence Soil Temperature in Drylands of Mediterranean Regions? *Water* **2018**, *10*, 1747, doi:10.3390/w10121747.
17. Delgado-Capel, M.J.; Egea-Cariñanos, P.; Cariñanos, P. Assessing the Relationship between Land Surface Temperature and Composition Elements of Urban Green Spaces during Heat Waves Episodes in Mediterranean Cities. *Forests* **2024**, *15*, 463, doi:10.3390/f15030463.
